# Multilineage commitment of Sca-1^+^ cells in reshaping vein grafts

**DOI:** 10.7150/thno.77735

**Published:** 2023-04-01

**Authors:** Zhichao Ni, Lingxia Lyu, Hui Gong, Luping Du, Zuoshi Wen, Hua Jiang, Hao Yang, Yanhua Hu, Bohuan Zhang, Qingbo Xu, Xiaogang Guo, Ting Chen

**Affiliations:** 1 Department of Cardiology, the First Affiliated Hospital, College of Medicine, Zhejiang University, Hangzhou, China.; 2 Department of kidney disease center, First Affiliated Hospital, School of Medicine, Zhejiang University, Hangzhou, 310003, Zhejiang, PR China.; 3 Alibaba-Zhejiang University Joint Research Center of Future Digital Healthcare, Hangzhou, China.

## Abstract

Vein graft failure remains a significant clinical problem. Similar to other vascular diseases, stenosis of vein grafts is caused by several cell lines; however, the sources of these cells remain unclear. The objective of this study was to investigate the cellular sources that reshape vein grafts. By analyzing transcriptomics data and constructing inducible lineage-tracing mouse models, we investigated the cellular components of vein grafts and their fates. The sc-RNAseq data suggested that Sca-1+ cells were vital players in vein grafts and might serve as progenitors for multilineage commitment. By generating a vein graft model in which the venae cavae from C57BL/6J wild-type mice were transplanted adjacent to the carotid arteries of Sca-1(Ly6a)-CreERT2; Rosa26-tdTomato mice, we demonstrated that the recipient Sca-1+ cells dominated reendothelialization and the formation of adventitial microvessels, especially at the perianastomotic regions. In turn, using chimeric mouse models, we confirmed that the Sca-1+ cells that participated in reendothelialization and the formation of adventitial microvessels all had a non-bone-marrow origin, whereas bone-marrow-derived Sca-1+ cells differentiated into inflammatory cells in vein grafts. Furthermore, using a parabiosis mouse model, we confirmed that non-bone-marrow-derived circulatory Sca-1+ cells were vital for the formation of adventitial microvessels, whereas Sca-1+ cells derived from local carotid arteries were the source of endothelium restoration. Using another mouse model in which venae cavae from Sca-1 (Ly6a)-CreERT2; Rosa26-tdTomato mice were transplanted adjacent to the carotid arteries of C57BL/6J wild-type mice, we confirmed that the donor Sca-1+ cells were mainly responsible for smooth muscle cells commitment in the neointima, particularly at the middle bodies of vein grafts. In addition, we provided evidence that knockdown/knockout of Pdgfrα in Sca-1+ cells decreased the cell potential to generate SMCs *in vitro* and decreased number of intimal SMCs in vein grafts. Our findings provided cell atlases of vein grafts, which demonstrated that recipient carotid arteries, donor veins, non-bone-marrow circulation, and the bone marrow provided diverse Sca-1+ cells/progenitors that participated in the reshaping of vein grafts.

## Introduction

Autologous vein grafts are surgical alternatives for revascularization; however, their patency rate is relatively low because of vein graft stenosis 1. Vein graft stenosis initiates during the injury of the donor veins, which is a consequence of the altered hemodynamic stress and surgical procedures 2. After the injury of vein grafts, leucocytes and smooth muscle cells (SMCs) accumulate in the intimal layer and accelerate hyperplasia 3. As a normal and necessary response to an altered environment, vein graft arterialization occurs as an adaptive process that is characterized by the thickening of the venous wall 4. However, vein graft failure occurs later, as a pathological process caused by a complicated vascular remodeling, including endothelial cell (EC) loss, associated inflammation, neointima formation, and atherosclerosis 5. After surgery, 20%-50% of vein grafts fail within 5 years, and by 10 years, 40% of the grafts are completely occluded 5. Within the neointima of vein grafts, the lesions at the perianastomotic regions and middle bodies are mainly characterized by focal and diffuse stenosis, respectively 6. Reendothelialization is pivotal in reducing the neointimal area and increasing the luminal area 7. Although this contention remains controversial, both circulating progenitor cells 8 and resident ECs 9 have been proposed as potential sources for EC regeneration. Recently, Wu et al. showed that mature SMCs in both recipient arteries and donor veins are important cell sources for neointima formation, and that the contribution of EndMT to neointima formation in vein grafts is marginal 10. However, the role and sources of other types of cells in vein graft remodeling remain unclear.

Sca-1 (Ly6a) was identified as a surface marker of hematopoietic stem cells 11. In recent decades, Sca-1^+^ cells were also shown to be distributed in the vessel wall 12-19 and participate in vascular remodeling. In 2004, abundant Sca-1^+^ cells were detected at the adventitia of the aorta and accelerated atherosclerosis by differentiating into SMCs 12. In 2018, a rare population of media SMCs expressing Sca-1 were also shown to be involved in the formation of atherosclerotic plaques 14. In turn, Wu et al. 10 and Majesky et al. 20 clearly demonstrated that a proportion of mature SMCs express Sca-1 and transform into stem-like cells during vascular remodeling. In addition to transforming into SMCs, the adventitial Sca-1^+^ cells also generate monocytes/macrophages, thus promoting vascular inflammation 16, 17. Tang et al. recently provided evidence that, in addition to contributing to neointma formation, Sca-1^+^ cells also play protective roles by restoring ECs 19 and/or SMCs 18 in injured vessels. Because of multilineage commitment, Sca-1^+^ cells may represent potential vascular progenitors that participate in vascular remodeling. Vein grafts feature a high rate of stenosis; however, the cell sources and underlying mechanisms remain unclear. Given the functions of Sca-1^+^ progenitors in vascular pathophysiology, we hypothesized that Sca-1^+^ cells from diverse sources exert different functions during the remodeling of vein grafts.

Although vein grafts can be harvested and investigated after failure, information regarding the cell sources and underlying mechanisms is not directly available. Therefore, several mouse models were used in this study to detect the cell sources and underlying mechanisms of vein graft stenosis. Using the vein graft murine model and the technique of single-cell RNA sequencing (scRNA-seq), we found that Sca-1^+^ cells/progenitors were potential sources of SMCs and ECs in vein grafts. Subsequently, to confirm the contribution of endogenous Sca-1^+^ cells to vein grafts, we generated inducible lineage-tracing mouse models. Our study demonstrated that Sca-1^+^ cells/progenitors from recipient carotid arteries participated in endothelium restoration, especially at perianastomotic regions of vein grafts, whereas those derived from non-bone-marrow circulation formed adventitial microvessels and those from donor veins mainly transformed into SMCs at the middle bodies of vein grafts. Finally, knockdown/knockout of Pdgfrα in Sca-1^+^ cells decreased the number of intimal SMCs in vein grafts, which was possibly a result of the decreased potential of Sca-1^+^ progenitors to differentiate into SMCs.

## Methods

### Animal Experiments

All the animal procedures were approved by the Institutional Animal Care and the Use Committee of Zhejiang University School of Medicine. All the animal procedures conformed to the guidelines from Directive 2010/63/EU of the European Parliament on the protection of animals used for scientific purposes. All the mice were bred with standard laboratory diet (Envigo, 7017C) in a 12-hour light and 12-hour dark condition at 22°C. In all the animal experiments, male and female mice were randomly selected at the age as indicated. Mice were euthanized with pentobarbital administration. (200 mg/kg, ip, once) (Sigma-Aldrich).

### Vein Graft Procedure

The procedures to perform vein graft were similar to that described previously 21. Briefly, the vena cava from donor mice was harvested and stored in heparin containing saline solution (100 U/mL). Recipient mouse was anesthetized by phenobarbital sodium (50 mg/kg) via intraperitoneal injection. The neck of mouse was incised from the midline, and then the right common carotid artery was carefully separated out and exposed. Both distal and proximal ends of the artery were clipped thoroughly. After the artery was dissected in the middle, a cuff in 1 mm diameter was placed over each end, respectively. The cuff was made from an autoclavable nylon tube with 0.63 mm in diameter outside and 0.5 mm inside (Portex Ltd., Hythe-Kent, UK). The vena cava from the donor mice was grafted between two ends of the artery. For each end, the vena cava covered carotid artery and was ligated tightly with an 8-0 suture. After the artery clippers were carefully removed, vigorous pulsation in the vein confirmed successful engraftment. Vein graft transplantation was performed between mice with C57BL/6J background including wild type C57BL/6J mice, Sca-1-CreER^T2^; Rosa26-tdTomato mice, chimeric mice, and Pdgfrα^flox/flox^; Sca-1-CreER^T2^; Rosa26-tdTomato mice as indicated. We divided the vein grafts into perianastomotic regions and middle bodies. The perianastomotic regions shown in our study were all from the proximal end of vein grafts. The whole lesion lengths surrounding the cuff were approximately 2mm and the lengths of the cuff were 1mm. Considering the suture is usually located at the middle of the cuff, the lengths of perianastomotic regions and middle bodies were approximately 0.5mm and 4mm, resulting in around 50 and 400 sections each with 10μm thickness.

### Single Cell Preparation and RNA Sequencing

In our scRNA-seq experiments, both male and female mice on C57BL/6J wild type background were randomly used. In our scRNA-seq, we collected the vein grafts 4 weeks after transplantation. The inferior venae cavae were firstly collected as the donor vein segments. Subsequently, the right common carotid arteries of recipient mice were mobilized. At the end, the donor vein segments from the donor mice were grafted between two ends of the carotid arteries. For isolation of single cell from carotid arteries and venae cavae as the controls, 8 mice aged 16 weeks were sacrificed. For preparation of vein graft mouse model, we used 16 mice (8 mice as recipients and donors, respectively) aged 12 weeks. The vein grafts were collected and single cells were isolated 4 weeks after the surgery. We flushed out the blood within the carotid arteries, the venae cavae, and the vein grafts by using intravascular PBS perfusion. Then the perivascular connective tissues were carefully peeled out and discarded. To obtain single cell suspensions, the cleaned vessels were digested with the solution consisting of papain (4 mg/ml), 1,4-Dithiothreitol (2 mg/ml), bovine serum albumin (1 ng/ml), Taurine (0.625 mg/ml) for 30 min at 37ºC, and then passed through a 40 μm cell strainer (Falcon, 352340). Subsequently, isolated cells were stained with DAPI and DRAQ5 to exclude the dead and unnucleated cells. Single live nucleated cell was then sorted by a BD FACS ARIA II Flow Cytometer (BD Biosciences). We isolated 7.8 x10ˆ5 cells (vein graft group), 1.5x10ˆ6 cells (carotid artery group) and 1.2x10ˆ6 cells (vena cava group), respectively. The viability of isolated cells in each group was >90%. Subsequently, we loaded 8,000 (vein graft group), 20,000 (carotid artery group) and 15,000 (vena cava group) cells according to standard 10x GemCode microfluidic chip-based technology.

Selected cells were prepared by using the Chromium single cell 3' regent Kits v2 from standard 10x GemCode microfluidic chip-based technology. Briefly, each single cell was partitioned separately into the nanoliter-scale Gel Bead in Emulsions (GEMs), in which the 16 nt 10x cell barcode and 10 nt unique molecular identifiers (UMI) were contained. After GEMs were broken and pooled cells were recovered, barcoded cDNA was amplified by PCR for library construction. The libraries were then sequenced using the Illumina pair-end sequencing. The cell ranger outputted 5162 cells with 17349 detected genes (vein graft group), 17644 cells with 17422 detected genes (carotid artery group) and 10517 cells with 18918 detected genes (vena cava group).

### ScRNA-seq Data Analysis

The sequencer's base cells file (BCL) was first demultiplexed into FASTQ file by using the cellranger mkfastq pipeline. Then the cellranger count pipeline was used to perform alignment, filtering, barcode counting, UMI counting and generate gene expression data in each single cell, as well as the feature-barcode matrix file. For further analysis of the single cell RNA data, Seurat (Version 3.1.1) R package 22 was used. Briefly, libraries data were read and stored in a Seurat object, which contained the information of each gene in each cell. The quality control metrics was then explored to filtered out the low-quality, multiplets and dying cells. Only cells expressing 200-5,000 genes and less than 10% mitochondrial counts were included into the further analysis. Selected data were log transformed by using the natural logarithm method log1p for normalization. The genes which exhibited high cell to cell variation were detected facilitating the biological signal investigation in the further analysis. Then a linear transformation scaling was applied to achieve a standard normal distribution dataset, so that the highly expressed genes did not dominate the result. To achieve the linear dimensional reduction, PCA was performed based on the PCA scores, in which the multiple gene information was combined. The top principle components with a strong enrichment of low p-value genes were calculated and chosen by using the JackStraw procedure 23. Subsequently, a K-nearest neighbor (KNN) graph was constructed based on Euclidean distance in the PCA space and group cells were clustered with similar gene expression pattern. The UAMP was used to visualize the data in low-dimensional plot. Both the positive and negative cell markers for all single cell clusters compared with other cells were identified using the FindAllMarkers function in the package. The differential gene expression between different clusters was investigated using the Wilcoxon rank sum test and genes with p-values <0.01 were regarded as differentially expressed. Biological function analysis of enrichment genes in cell clusters was performed by using the Metascape method 24.

### Pseudotime Trajectory Analysis

For the pseudotime trajectory analysis, Monocle R package was used 25. Top 2000 genes which exhibited highest cell to cell variation defined in the Seurat package described above were used for further gene ordering. Subsequently, the machine learning technique DDRTree was used for data dimension reduction, which projected the data into a lower dimensional space. Then the trajectory path which described the cell transition from one state to another was established by cell ordering. The pseudotime value of one cell was the distance it had to travel back to the origin point. Then the expression change of selected genes could be tested during the calculated biological process in a pseudotime manner. To detect the genes which showed a similar kinetic transitional trend, the changes of gene expression were constructed and visualized in the heatmap.

### Sca-1(Ly6a)-CreER^T2^, Pdgfrα^flox/flox^ and Rosa26-tdTomato reporter Mice

Sca-1(Ly6a)-CreER^T2^; Rosa26-tdTomato mice were designed and generated in accordance with genOway company. Homologues sequences of Ly6a gene from C57BL/6J mice were cloned resulting in a long upstream fragment (4.05 kb) and a short downstream fragment (2.12 kb). The long homology arm contained exon 1, exon 2 non-coding sequences and neighbouring intron sequences, whereas the short arm contained exon 2 coding sequences, exon 3 and neighbouring intron sequences. A cDNA encoding CreER^T2^ coding sequence and frt sites flanked neomycin cassette was inserted in frame of the Ly6a ATG start sequence. CreER^T2^ cDNA encoded the Cre recombinase along with ER^T2^ a mutant estrogen receptor. This mutant estrogen receptor is sensitive to tamoxifen rather than endogenous estrogen. NEO sequence encoded protein was served for positive selection by adding geneticin (G418). The NEO selection sequence was later deleted by applying Flp-frt based system. The targeting vector contained 5' long homology region, CreER^T2^ coding sequence, NEO positive selection sequence flanked by frt sites and 3' short homology region. The targeting vector also contained a diphtheria toxin (DTA) cassette that was used for negative vector selection at upstream of 5' long arm. The quality of targeting vector was controlled by identifying the gene sequences described above. The Ly6a^+/CreER^ targeting vector was then linearized by restriction digestion followed by phenol purification and ethanol precipitation. Linearized targeting vector was transfected into embryonic stem cells according to genOway's electroporation procedures. Positive selection was based on adding the G418. The G418 resistant colonies were then selected and amplified for storage or further genomic DNA identification. Further genomic screening was conducted by PCR and southern blot. Selected embryonic stem cells were then injected into blastocysts to produce highly chimeric mice carrying the recombined locus. Chimeric male mice were crossed with Flp-deleter female mice for deleting frt flanked NEO selection sequence. Resulting Ly6a^+/CreER^ mice were then crossed with C57BL/6J mice for germline transmission to generate the stable line for further use. PCR primers (Neo deleted knock in recombination allele, forward: 5'-CAAGCTGGAGTGCAGTGGCACAATC-3', reverse: 5'-CCATTGACTTGGCATCTGGGGAG-3'; Wild type allele, forward: 5'-CCTGTGCTTTATGACTCTTGGTAG-3', reverse: 5'-AGGTTCTGTGGCAGAGATACTGAG-3') were used to test the target allele across all the target DNA containing inserted cassette. Rosa26-tdTomato reporter mice 26 (ROSA26Sor^tm9(CAG-tdTomato)Hze^/J, 007909) were purchased from the Jackson Laboratory, USA and were crossed with C57BL/6J mice for expansion. PCR primers (Rosa26-tdTomato recombination allele, forward: 5'-CTGTTCCTGTACGGCATGG-3', reverse: 5'-GGCATTAAAGCAGCGTATCC-3'; Wild type allele, forward: 5'-AAGGGAGCTGCAGTGGAGTA-3', reverse: 5'-CCGAAAATCTGTGGGAAGTC-3') were used to test the target allele. Pdgfrα^flox/flox^ mice (C57BL/6-Pdgfra^tm1(flox)Smoc^, NM-CKO-200203) were purchased from Shanghai Model Organisms Center, Inc. PCR primers (Recombination allele, forward: 5'-CAGACATTACTCCAAAGTTAGGCACC-3', reverse: 5'-TCTTTCCTCACTGCCACCCTCT-3') were used to test the target allele. Sca-1-CreER^T2^; Rosa26-tdTomato mice were generated by crossing the Sca-1-CreER^T2^ knock in and Homozygous Rosa26-tdTomato reporter mice. Pdgfrα^flox/flox^; Sca-1(Ly6a)-CreER^T2^; Rosa26-tdTomato mice were generated by crossing the Pdgfrα^flox/flox^ and CreER^T2^; Rosa26-tdTomato mice with further appropriate genotyping. 5 pulses of tamoxifen were given to the mice as indicated to conditionally induce gene recombination and therefore tdTomato reporter gene expression and/or *Pdgfrα* gene deletion. We collected vessels at least 1 week after the last dose of tamoxifen according to different surgical procedures as described.

### Polymerase Chain Reaction

Genotypes of Sca-1-CreER^T2^; Rosa26-tdTomato and Pdgfrα^flox/flox^; Sca-1(Ly6a)-CreER^T2^; Rosa26-tdTomato mice were identified by polymerase chain reaction (PCR). Genomic DNA from murine tail was collected and amplificated when mixed with target primers and Taq DNA polymerase. Resulting PCR products were put on 2% agarose gel (Invitrogen, 16500-500) for separation. Target bands were detected by using BioSpectrum Imaging system.

### Bone Marrow Transplantation

The procedures used for creating chimeric mice were similar to that described previously 27, 28. Briefly, donor mice were sacrificed; the femurs and tibias were removed aseptically. Donor bone marrow cells were flushed out from marrow cavities and then passed through the 40-μm cell strainers to eliminate the blood clot. Cells were collected and stored in serum-free DMEM (ATCC, 30-2002) for transplantation. The bone marrow of recipient mice were destroyed by a lethal dose of X-ray irradiation (900rads) as described previously 27. 6 hours after the bone marrow irradiation, 5×10^6^ donor bone marrow cells were injected via tail vein to the recipient mice.

### Flow Cytometry Analysis

Bone marrow cells were flushed out from the femurs and tibias of mice. The blood was collected venipuncture from *vena cava*. Both bone marrow cells and blood were incubated with red blood cell lysis buffer (eBioscience, 00-4333) to remove red blood cells. All prepared cell samples were kept on ice before flow cytometric analysis. All the samples were collected by BD ACCURI C6 flow cytometer and all the data were analysed using FlowJo software.

### Parabiotic Procedure

The chimeric and the C57BL/6J mouse were both anesthetized by phenobarbital sodium (50 mg/kg). The dorsal and ventral skins were incised to expose the elbow and the knee joints. After the respective joints (elbow to elbow, knee to knee) from both mouse lines were sutured together, the skin was closed by manual suture. In the mouse model, the mice shared a parabiotic circulation due to formation of collateral vessels.

### Immunofluorescence and Immunohistochemical Staining

In the present study, vessels and other organs including vein grafts, vena cavae, carotid arteries, aortas, lungs, spleens and kidneys were s separately collected at the indicated time points. Vessels along with other organs were separately washed by PBS and rinsed in 4% paraformaldehyde (PFA, Santa Cruz, sc-281692) at 4°C for 2-3 h for fixation. Then the vessels and other organs were put into 30% sucrose solution (BDH, 102747E) overnight at 4°C for dehydration, and embedded in OCT at -80°C. Sections at 10 μm thickness were prepared after cryosectioning using CryoStar Cryostat (Thermo Scientific). Cryosections were permeabilized and blocked by 5% donkey serum and 0.1% Triton X-100 for 1 h at room temperature. The cryosections were stained with primary antibodies overnight and afterwards Alexa Fluor-conjugated secondary antibodies (Invitrogen, 1:500) were put on the section for 1 h. At the end, 4',6-Diamidino-2-Phenylindole, Dilactate (DAPI, Molecular Probe, D1306) was put on the sample for 10 min to visualize the nuclei. For whole mount fluorescence images staining of vein grafts, We used the protocols similar to previous study 29. We firstly fixed the grafts with 4% PFA overnight. Subsequently, the grafts were immersed in CUBIC-L solution (10% N-butyldiethanolamine (TCIchemicals, B0725) and 10% Triton X-100 38 in ddH2O) at 37°C for 2 days. The resulted grafts were then incubated with primary antibodies and Alexa Fluor-conjugated secondary antibodies, each for 1day. At the end, the grafts were put into the CUBIC-R solution (45% (antipyrine (TCIchemicals, D1876), 30% nicotinamide (TCIchemicals, N0078), 0.5% N-butyldiethanolamine in ddH2O) for transparency. Leica (TCS SP5) confocal microscopy was used to capture the images. For images quantification, images from each section were analysed by Image J 30 and the cell numbers were counted in each section as indicated. Primary antibodies used in this study included Sca-1 (Abcam, ab51317, 1:200), tdTomato (Rockland, 600-401-379, 1:500), α-SMA (Sigma, A5228, 1:500), SM22 (Abcam, ab14106, 1:50), Calponin (Abcam, ab46794, 1:50), CD31 (BD Pharmingen, 553370), VE-Cadherin (Abcam, ab33168, 1:50), von Willebrand factor (VWF) (Abcam, ab6994, 1:50). Counterpart primary antibodies of IgG control plus secondary antibodies were used as the negative control of immunofluorescence.

### Sca-1^+^ Cell Culturing, Transfection and Differentiation

Sca-1^+^ vascular cells/progenitors were collected from the aortic adventitia of C57BL/6J wild type mice as previously described 12. Briefly, the aortic adventitia was harvested and flattened on a flaks coated with 0.04% gelatin (Sigma, G1393) and cultured in complete stem cell culture medium, consisting DMEM (American Type Culture Collection, 30-2002, USA), 10% ES cell qualified fetal bovine serum (Embriomax, ES-009-B, USA), 10ng/ml of leukemia inhibitory factor (Merck Millipore, LIF1050, USA), 0.1 mM 2-mercaptoethanol (Sigma, M3148, USA) and 100 U/mL Penicillin-Streptomycin solution (Cienry, CR-15140, China). Cells grown from the aortic adventitia were then sorted with sca-1 microbeads (Miltenyi Biotec, 130-123-124) following manufacturer's instructions. The sorted cells were cultured with complete stem cell culture medium and passaged every 3 days. For cell differentiation experiments, we treated the cells without (as a control) or with 10ng/ml TGFβ1 (R&D systems, 7666-MB-005, USA) along with the cell differentiation culture medium which consisted of 10% ES cell qualified fetal bovine serum (Embriomax, ES-009-B, USA), 0.1 mM 2-mercaptoethanol (Sigma, M3148, USA) and 100 U/mL Penicillin-Streptomycin solution (Cienry, CR-15140, China). We purchased siRNAs including si*NC* (forward: 5'-UUCUCCGAACGUGUCACGUTT-3', reverse: 5'-ACGUGACACGUUCGGAGAATT-3') and si*Pdgfrα* (forward: 5'-GGAUGAGAGUGAGAUCGAATT-3', reverse: 5'-UUCGAUCUCACUCUCAUCCTT-3') from the company (Hanbio, China). Sca-1^+^ cells were subsequently transfected with the siRNAs. Briefly, the cells were transfected with 20 μM siRNA, Opti-MEM (Gibco, 31985-070, USA), and RNAiMAX reagent (Invitrogen, 13778150, USA). After 6h incubation, the medium was changed to differentiation culture medium without (as a control) or with 10ng/ml TGFβ1. After 72h culture, the cells were harvested for further analysis.

### Western Blot Analysis

Total protein was extracted using RIPA Lysis Buffer (Beyotime, P0013B, China) and quantified by Enhanced BCA Protein Assay Kit (Beyotime, P0010S, China). The protein samples were then electrophorized and transferred on PVDF membranes (Millipore, IPVH00010, USA) using eBlot L1 Fast Wet Transfer System (GeneScript, L00686C, Singapore). Membranes with proteins were blocked in Blocking Buffer (Beyotime, P0252, China) for 1 hour and incubated overnight at 4˚C with primary antibodies. Subsequently, the membranes were incubated with HRP-conjugated secondary antibody at room temperature for 1 hour. The enhanced chemiluminescence kit (Millipore, WBKLS0500, USA) was utilized to visualize the bands in the ChemiDoc XRS+ System (Bio-Rad, USA). The integrated density of the bands was quantified by Fiji software. Primary antibodies used in the study included α-SMA (Sigma 14395-1-AP 1:2000), SM22 (Abcam ab14106 1:2000), Calponin (Abcam ab46794 1:1000).

### Bulk RNA Preparation, Sequencing and Analysis

Total RNA from Sca-1^+^ vascular cells/progenitor was extracted using TRIzol regent (Invitrogen, CA, USA) according to the manufacturer's instructions. Then the libraries were constructed using VAHTS Universal V6 RNA-seq Library Prep Kit according to the manufacturer's instructions. The transcriptome sequencing and analysis were conducted by OE Biotech Co., Ltd. (Shanghai, China). The libraries were sequenced on an Illumina Novaseq 6000 platform and 150 bp paired-end reads were generated. Bulk RNA-seq data from bone marrow derived Sca-1^+^c-Kit^+^Lin^-^ cells (LSKs) were downloaded from public dataset GSE216794. Q value < 0.05 and foldchange > 10 or foldchange < 0.1 was set as the threshold for significantly differential expression gene (DEGs). Significant genes that are related to vascular development were drew in the volcano plot. Biological function analysis of enrichment genes in each cell population was performed by using the Metascape method 24.

### Statistics Analyses

All data are presented as mean ± standard error of the mean (SEM). The number of animals used in the study is shown in each figure legend. GraphPad Prism 8 software was used for statistical analysis. All data were analyzed with and passed normality and variance tests. Data from two groups were analyzed by unpaired t-test, and those among multiple groups were compared using one-way ANOVA with Dunnett's or Tukey's post hoc tests as indicated in the figure legend. *P*<0.05 was thought to be statistically significant.

### Data Availability

The data of this study are available from the corresponding author upon reasonable request. Single cell RNA sequencing (scRNA-seq) data of current study have been made publicly available GEO repository and can be accessed at GSE140879.

## Results

### Sc-RNAseq-Based Identification of the Cell Types in Vein Grafts

We first constructed a vein graft mouse model in which the venae cavae were isolated and isografted adjacent to the carotid arteries of recipient animals. Both donor and recipient mice were in the C57BL/6J genetic background. Male and female mice were randomly used in our study. Images of hematoxylin and eosin (H&E)-stained sections showed that freshly harvested venae cavae were composed of one layer of ECs, one-to-two layers of SMCs, and an adventitial layer. As described previously 31, after vein grafting, the donor veins were damaged, followed by massive mononuclear cell infiltration of all vein graft layers. Similar to that reported by other studies 10, 32, the intimal area and the thickness of vessel walls were increased, whereas the luminal area was decreased in a time-dependent manner. By 4 weeks after the surgeries, both the thickness of vessel walls and the intimal area were remarkably increased. Therefore, we collected the vein grafts 4 weeks after surgery and found multiple cellular layers together with abundant extracellular matrix in both the intima and the adventitia (Figure S1A-B). Similar to the previous report by Wu et al. 10, the vein grafts exhibited both focal stenotic lesions with a smaller luminal area at perianastomotic regions and diffused lesions at the middle bodies.

**Figure 1:**
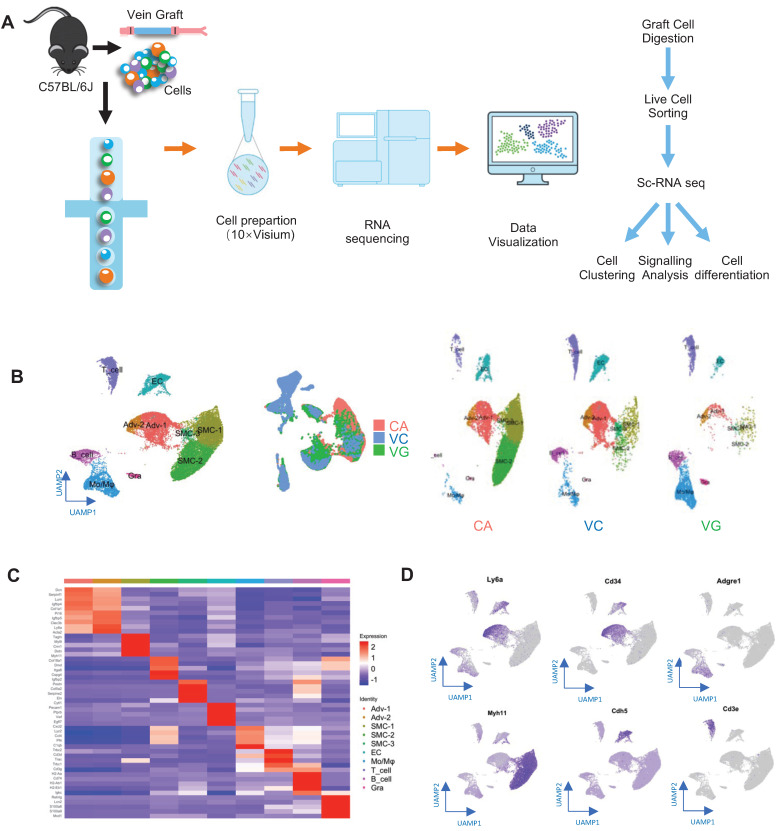
Identification of Vein Graft Components. (A) Schematic showing vessel preparation (CA, VC and VG) for scRNA-seq and data analysis. Images shown are including n=8 separate vessels or grafts. (B) Plots representing gene expression using UAMP showing major cell populations of vein graft. Colour coded cell populations in either CA, VC or VG. (C) Heatmap demonstrating the top 5 gene markers among cell clusters. (D) Feature plot representative of gene expression of Ly6a, Cd34, Adgre1, Myh11, Acta2 and Cd3e. Abbreviations: Adv, adventitial cell; CA, carotid artery; EC, endothelial cell; Mo/Mø, monocyte/macrophage; scRNA-seq, single cell RNA sequencing; SMC, smooth muscle cell; UAMP, uniform manifold approximation and projection; VC, vena cava; VG, vein graft.

Subsequently, to obtain single-cell suspensions for single-cell RNA sequencing (scRNA-seq), vein grafts were collected and digested (Figure 1A). We also collected venae cavae and carotid arteries, to compare their transcriptomic differences with vein grafts. For the isolation of single cells from carotid arteries and venae cavae, eight mice aged 16 weeks were sacrificed. For the preparation of the vein graft mouse model, eight grafts were generated, in which both the donor and the recipient mice were aged 12 weeks. We collected the vein grafts and isolated single cells 4 weeks after the surgeries. The nucleated live single cells were sorted and distributed with individual barcodes in GEMs that contained UMIs (Figure S1C). On average, each cell yielded 29,677 reads. We performed unsupervised clustering using the Seurat package, to identify cell clusters; the results were visualized by uniform manifold approximation and projection (Figure 1B). Cell types were identified according to their markers, as labeled (Figure 1B-C). Cell clusters including ECs, SMCs, and adventitial mesenchymal cells were identified in the murine vessels (Figure 1B-D). In addition, our study also detected leucocytes, including macrophages, lymphocytes, and granulocytes. These results indicated that both vascular cells (including ECs, SMCs, and adventitial mesenchymal cells), and leucocytes participated in vein graft remodeling. Our subsequent analysis focused on the vascular cells.

### Identification of EC Progenitors in Vein Grafts Using Sc-RNAseq

**Figure 2:**
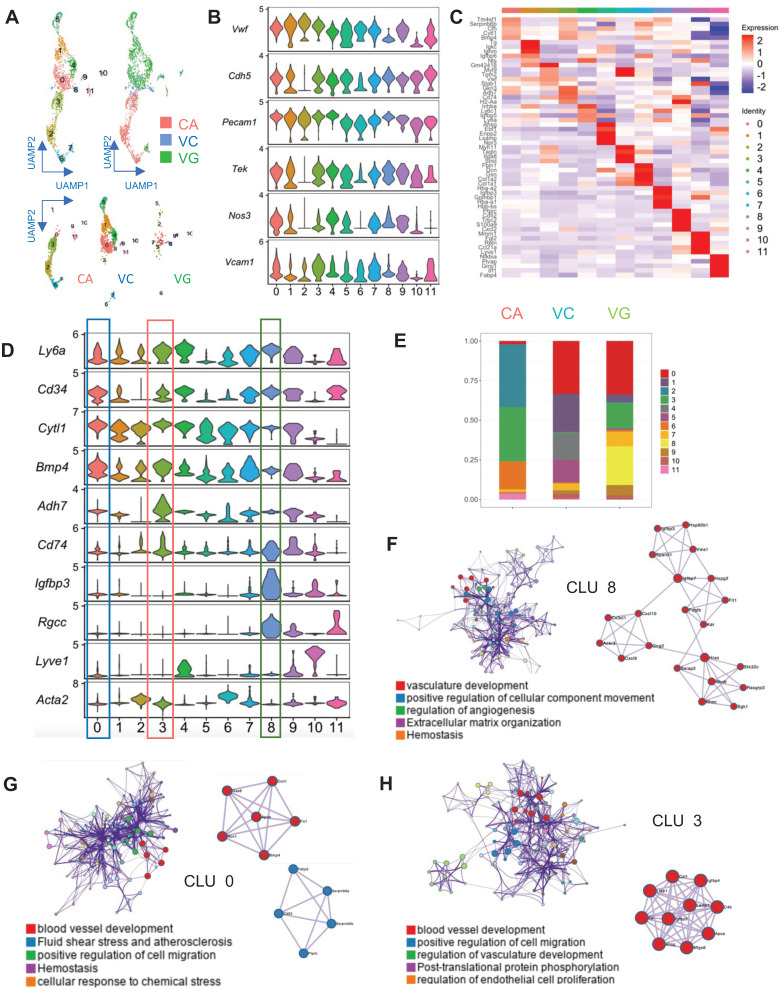
Analysis of ECs in ScRNA-seq of Vein Grafts. (A) Scatter plot showing EC clusters. Colour coded cell populations in either CA, VC or VG. (B) Violin plot displaying genetic expression of EC markers in the subpopulations. (C, D) Heatmap and Violin plot demonstrating the markers among EC clusters. (E) Bar chart showing the percentage of cell types in distinct groups. (D) Heatmap of specific marker genes in each EC cluster. (E) Violin plot indicating the EC markers in the whole EC populations. (F-H) Biological function analysis of enrichment genes in Cluster 8, Cluster 0, Cluster 3. Abbreviations: CA, carotid artery; CLU, cluster; EC, endothelial cell; UAMP, uniform manifold approximation and projection; VC, vena cava; VG, vein graft.

EC activation and regeneration are common processes in vein grafts. Therefore, we examined the EC cluster in the sc-RNAseq data; this cell cluster consisted of 541 ECs. We further divided the cluster into 12 subpopulations (Figure 2A). All subclusters expressed canonical EC markers, including *Pecam1, Vwf*, and *Cdh5* (Figure 2B). In turn, they also displayed variable genetic patterns (Figure 2C). We calculated the proportion of each subcluster (Figure 2D), and found that cluster 0 (*Cytl1 and Bmp4*) was a major component of ECs in the venae cavae, whereas cluster 3 (*Adh7 and Cd74*) was the main EC population in the carotid arteries (Figure 2D). Cluster 0 and cluster 3 also accounted for 27.8% and 23.3% of ECs in the vein grafts, respectively (Figure 2E). These results suggest that the cells from both carotid arteries and veins donate ECs to vein grafts. Interestingly, cluster 8 (*Igfbp3* and *Rgcc*) accounted for a high percentage (26.2%) of all ECs detected in the vein grafts, but was nearly absent among those present in both the carotid arteries and the venae cavae (Figure 2E). This result indicates that, in addition to arteries and veins, circulating cells may serve as a third source of ECs in vein grafts. An analysis of the gene ontology biological processes suggested functions for clusters 0/3/8 in angiogenesis and vascular development (Figure 2F-H). Moreover, we found that all of the EC subclusters described above displayed a high expression level of Sca-1 (Figure 2D). Collectively, these results suggest that Sca-1^+^ cells from variable sources form ECs in vein grafts; however, the spatial sources remained unknown.

### Sca-1(Ly6a)-CreER^T2^; Rosa26-tdTomato Lineage-tracing Mouse Model

Because of the high heterogeneity of Sca-1^+^ cells, as shown in the sc-RNAseq experiment, we continued to examine the role of endogenous Sca-1^+^ cells *in vivo*. To trace the fate of Sca-1^+^ cells, we generated an Sca-1(Ly6a)-CreER^T2^; Rosa26-tdTomato lineage-tracing mouse model (Figure S2A-C). Sca-1-CreER^T2^; Rosa26-tdTomato mice were administered 5 pulses of tamoxifen (Figure S2C), and vessels (including aortas, carotid arteries, and venae cavae) and other organs were collected 1 week after the administration of the last dose of tamoxifen. Using flow cytometry, we showed that tdTomato labeled cells from both the bone marrow and blood (Figure S2D-E). These results indicated the successful establishment of the lineage-tracing mouse model. Sca-1^+^ cells were detected in organs such as the lung 33, spleen 34, and kidney 35. Therefore, we performed immunostaining on sections of these organs to test the specificity of our labeling system. After the administration of pulses of tamoxifen to the mice, the co-expression of tdTomato and Sca-1 antibodies was observed in all organs (Figure S3A), which indicated the successful labeling of Sca-1-CreER^T2^; Rosa26-tdTomato mice. In contrast, no tdTomato^+^ cells were observed in the negative control animals, which did not receive the tamoxifen pulses (Figure S3A); this result indicated the absence of leakiness in the labeling system. Subsequently, we detected tdTomato expression in vascular sections using the lineage-tracing system described above. We analyzed aortas, venae cavae, and carotid arteries with average lengths of 40, 10, and 10 mm, respectively. The vessels were cut into sections with a thickness of 10 μm. Our laboratory previously demonstrated the presence of abundant Sca-1^+^ cells in aortas 12. A consistent result was obtained in the current study, as tdTomato^+^ cells were distributed in the intima and the adventitia of aortas (Figure S3B, S3C). Similarly, in carotid arteries, we found that tdTomato^+^ cells were sporadically spread in both the intimal and the adventitial layers (Figure S3D, S3E). Although progenitors have also been shown to be vital in venous disease 36, no studies have addressed the existence of Sca-1^+^ cells in the venous system*.* Therefore, we investigated whether Sca-1^+^ cells were present in venae cavae*.* Techniques including vascular longitudinal sectioning, *en face* staining 37, and cross-sectioning were applied to localize Sca-1^+^ cells. We found that tdTomato^+^ cells were mainly located at the adventitia (S3H) and sporadically distributed at the intima (Figure S3B-C, S3H).

### Sca-1^+^ Cells from the Recipient Regenerated the Endothelium at Perianastomotic Regions

**Figure 3:**
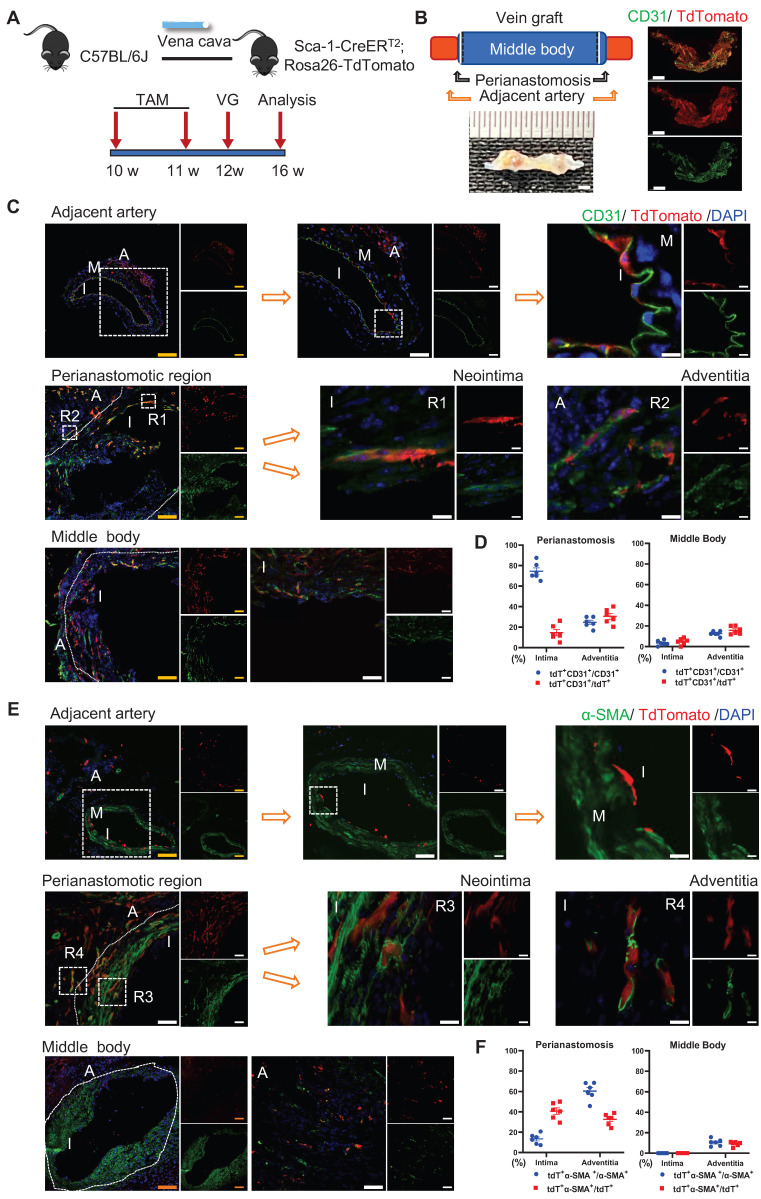
Recipient Sca-1+ Cells Mainly Regenerating Vascular Cells at the Perianastomotic Regions of Vein Grafts. (A) Schematic showing vein graft model (n=6 experiments). After Ly6a-CreERT2; Rosa26-TdTomato mice received tamoxifen treatment, venae cavae from C57BL/6J mice were transplanted adjacent to the carotid arteries of Ly6a-CreERT2; Rosa26-TdTomato mice. Grafts were collected 4 weeks after the surgeries. (B) Representative whole-mount images showing TdTomato and CD31 labeling in vein grafts with adjacent arteries (Scale bars: 1 mm). Images shown are representative of n=3 separate grafts. (C) Vein graft sections at perianastomotic regions and middle bodies, along with adjacent carotid arteries, were stained with TdTomato and CD31 as indicated (Scale bars: 100 μm in yellow, 50 μm in white, and 10 μm in enlarged image). Images shown are representative of n=6 separate grafts. (D) The panel showing percentage of TdTomato expression in CD31+ ECs or CD31 expression in TdTomato+ cells. All the data represent mean ± SEM, n=6 per group. (E) Vein graft sections at perianastomotic regions and middle bodies, along with adjacent carotid arteries, were stained with TdTomato and α-SMA as indicated (Scale bars: 100 μm in yellow, 50 μm in white, and 10 μm in enlarged image). Images shown are representative of n=6 separate grafts. (F) The panel representing quantification percentage of cells expressing TdTomato in α-SMA+ SMCs, or cells expressing α-SMA in TdTomato+ cells. All the data represent mean ± SEM, n=6 per group. Abbreviations: A, adventitia; α-SMA, α-Smooth Muscle Actin; I, intima; M, media; TAM, tamoxifen; VG, vein graft.

The sc-RNAseq data reported indicated that the Sca-1^+^ cells/progenitors that formed ECs in vein grafts may be derived from different sources, including carotid arteries, venae cavae, and the circulation. We subsequently designed several mouse models to examine the roles of cells from these sources in vein grafts. First, we tested the role of the recipient Sca-1^+^ cells by combining the linage-tracing and vein graft mouse models. In the clinical setting, recipient cells originate from a combination of arterial and circulatory sources. In the combined mouse model, Sca-1-CreER^T2^; Rosa26-tdTomato mice were first pulsed with tamoxifen, and then the venae cavae from the C57BL/6J mice was transplanted adjacent to the carotid arteries of the Sca-1-CreER^T2^; Rosa26-tdTomato mice (n = 6 experiments) (Figure 3A). Because the Sca-1-CreER^T2^ transgene was inserted into Chr. 15 without affecting the sex chromosomes, both male and female mice were randomly used in our study. We collected the vein grafts 4 weeks after the surgeries. Whole-mount fluorescence images showed that the grafts were strongly labeled by tdTomato; moreover, a massive amount of tdTomato^+^ CD31^+^ cells were observed across all vein grafts (Figure 3B). Subsequently, we cut the vein grafts into sections with a thickness of 10 μm. The vein grafts exhibited both focal stenotic lesions at perianastomotic regions and diffuse lesions at the middle bodies, both of which showed intimal hyperplasia and expanded adventitia (Figure 3C, 3E).

We observed that massive quantities of tdTomato^+^ cells accumulated across all vein grafts, indicating that Sca-1^+^ cells originating from the recipient were involved in vein graft remodeling (Figure 3C, 3E). In these vein grafts, we observed that tdTomato^+^ ECs regenerated the endothelial layer and formed adventitial microvessels (Figures 3C and S4A). We analyzed sections of the vein grafts according to anatomical location. At perianastomotic regions, 74.50% ± 3.33% and 24.70% ± 2.08% of CD31^+^ ECs were labeled by tdTomato in the intimal and adventitial microvessels, respectively. In turn, at the middle bodies, tdTomato was expressed in 3.45% ± 1.03% and 12.70% ± 0.96% of CD31^+^ ECs in the intima and the adventitia, respectively (Figure 3D).

SMC accumulation is a hallmark of the neointima, and Sca-1^+^ cells/progenitors have been suggested as a potential source of SMCs 12, 18. We found that SMCs were the major cell type in the neointima, representing approximately 63% and 88% of the cells detected at the perianastomotic regions and middle bodies, respectively (Figure S4E). The percentages of SMCs were also quantified at the adventitia, which were 3.3% at the perianastomotic regions and 0.5% at the middle bodies (Figure S4E). We next investigated whether the recipient-derived Sca-1^+^ cells participated in SMC commitment in vein grafts. Within the intima, we found that tdTomato^+^ cells expressed the SMC markers α-SMA, SM22, and Calponin (Figures 3E and S4B-C). Statistically, we found that 13.43% ± 2.06% of α-SMA^+^ SMCs were derived from tdTomato^+^ cells at the perianastomotic regions, whereas no tdTomato^+^ SMCs were observed at the middle bodies (Figure 3F). SMCs were also shown to contribute to adventitial remodeling 38. In our study, we observed an increase in the number of SMCs in the adventitia of vein grafts. Importantly, 60.33% ± 3.48% and 10.50% ±1.40% of the α-SMA^+^ SMCs were labeled by tdTomato in the perianastomotic regions and middle bodies, respectively (Figure 3F). Collectively, these results suggest that recipient Sca-1^+^ cells are vital players in reendothelization, the commitment of neointimal SMCs, and the formation of microvessels mainly at the perianastomotic regions of vein grafts.

### Bone-marrow-derived Sca-1^+^ Cell-induced Inflammation

Our study demonstrated that recipient Sca-1^+^ cells generated ECs and participated in SMC formation in vein grafts; however, the exact origin of these cells remained elusive. The bone marrow has been regarded as a potential source of vascular SMCs 39-41 and/or ECs 8, 42, 43. Therefore, we divided the whole Sca-1^+^ cells of the recipient mice into bone-marrow- and non-bone-marrow-derived groups and tested their respective functions in vascular remodeling.

To verify the function of bone-marrow-derived Sca-1^+^ cells, a chimeric mouse model was created by transplanting the bone marrow cells from Sca-1-CreER^T2^; Rosa26-tdTomato mice into C57BL/6J mice that had been irradiated with X-rays (Figure S5A). After the administration of tamoxifen pulses, successful bone-marrow reconstruction was confirmed by detecting tdTomato signals in the bone marrow and in the peripheral blood using flow cytometry (Figure S5B). Subsequently, venae cavae were transplanted from C57BL/6J mice and introduced adjacent to the carotid arteries of these chimeric mice (n = 6 experiments) (Figure S5A). In this mouse model, tdTomato signals were still observed in the vein grafts (Figure S5C-E). However, no α-SMA^+^ (Figure S5C) or CD31^+^ (Figure S5D) cells were labeled with tdTomato in either the intima or the adventitia. These results indicated that bone-marrow-derived Sca-1^+^ cells do not form ECs or SMCs in vein grafts; rather, CD11b^+^ tdTomato^+^ cells were observed in both the neointima and the adventitia (Figure S5E). Collectively, these data suggest that bone-marrow-derived Sca-1^+^ cells differentiate into inflammatory cells, but not SMCs or ECs, in vein grafts.

### Non-BM-derived Circulatory Sca-1^+^ Cells Displayed Novel Functions

**Figure 4:**
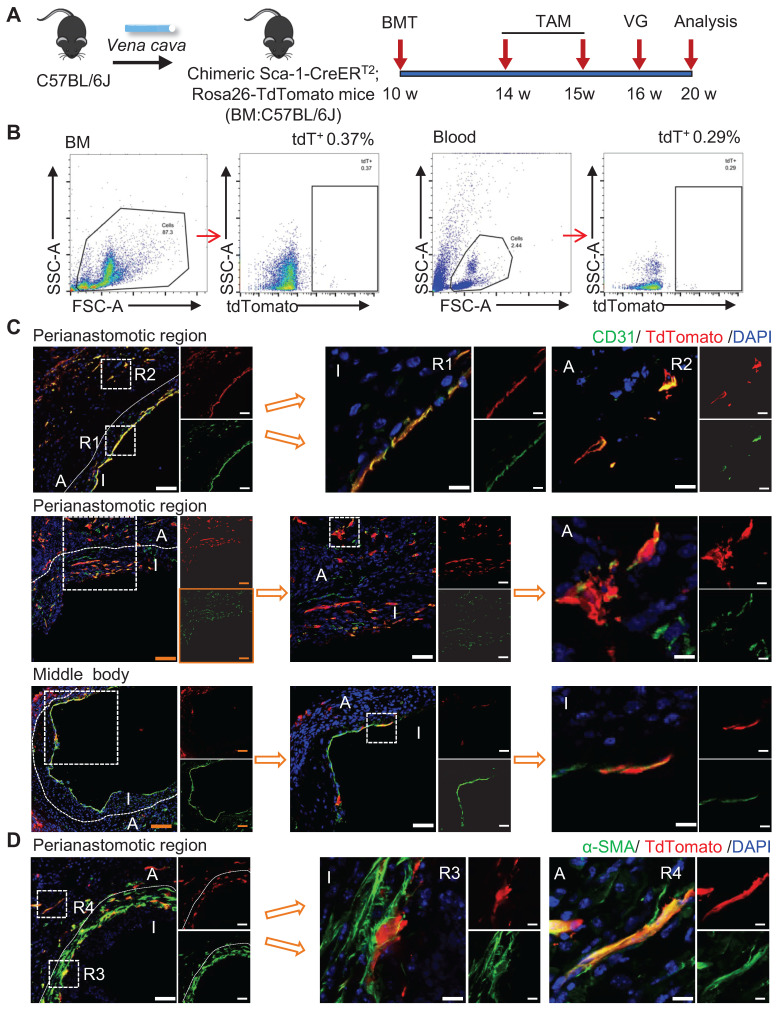
Non-bone Marrow Sca-1+ Cells Mainly Involved in EC Restoration and Microvessels Formation at the Perianastomotic Regions of Vein Grafts. (A) Strategy for chimeric mouse model in which bone marrow cells from C57BL/6J mice were transplanted to irradiated Ly6a-CreERT2; Rosa26-TdTomato mice. Four weeks after bone marrow transplantation, pluses of tamoxifen were given to the chimeric mice. Subsequently, venae cavae from C57BL/6J mice were transplanted adjacent to the carotid arteries of the chimeric mice. Grafts were collected 4 weeks after the surgeries. (B) Representative flow cytometric analysis of TdTomato+ cells from bone marrow and blood in the chimeric mice(n=6). (C) Vein graft sections at perianastomotic regions and middle bodies were stained with TdTomato and CD31 as indicated (Scale bars: 100 μm in yellow, 50 μm in white, and 10 μm in enlarged image). Images shown are representative of n=6 separate grafts. (D) Vein graft sections at perianastomotic regions were stained with TdTomato and α-SMA as indicated (Scale bars: 50 μm, and 10 μm in enlarged image). Images shown are representative of n=6 separate grafts. Abbreviations: A indicates adventitia; α-SMA, α-Smooth Muscle Actin; BMT, bone marrow transplantation; I, intima; R1-4, region 1-4; TAM, tamoxifen; VG, vein graft.

To verify the role of non-bone-marrow-derived Sca-1^+^ cells in vein graft remodeling, another chimeric mouse model was generated by transplanting the bone-marrow cells from C57BL/6J mice to irradiated Sca-1-CreER^T2^; Rosa26-tdTomato mice (Figure 4A). Similar to the protocol described above, these chimeric mice were pulsed with tamoxifen (Figure 4A). Using flow cytometric, we revealed that bone-marrow cells were successfully reconstituted because of the near-total absence of a tdTomato signal in both blood and bone-marrow samples (Figure 4B). We collected vessels, including aortas and carotid arteries, and found that tdTomato^+^ cells were distributed in the intima and the adventitia (Figure S6A-B). Subsequently, venae cavae were transplanted from C57BL/6J mice to a location adjacent to the carotid arteries of the chimeric mice (n = 6 experiments). Four weeks after the surgeries, tdTomato^+^ cells were observed mainly at the perianastomotic regions of the vein grafts (Figure 4C-D). Furthermore, some tdTomato^+^ cells exhibited EC markers, which participated in EC regeneration and microvessel formation (Figures 4C and S6C). In addition, we observed that part of the tdTomato^+^ cells were stained for α-SMA (Figure 4D). These data suggest that the recipient Sca-1^+^ cells that formed ECs and SMCs in the vein grafts stemmed from non-bone-marrow sources.

**Figure 5:**
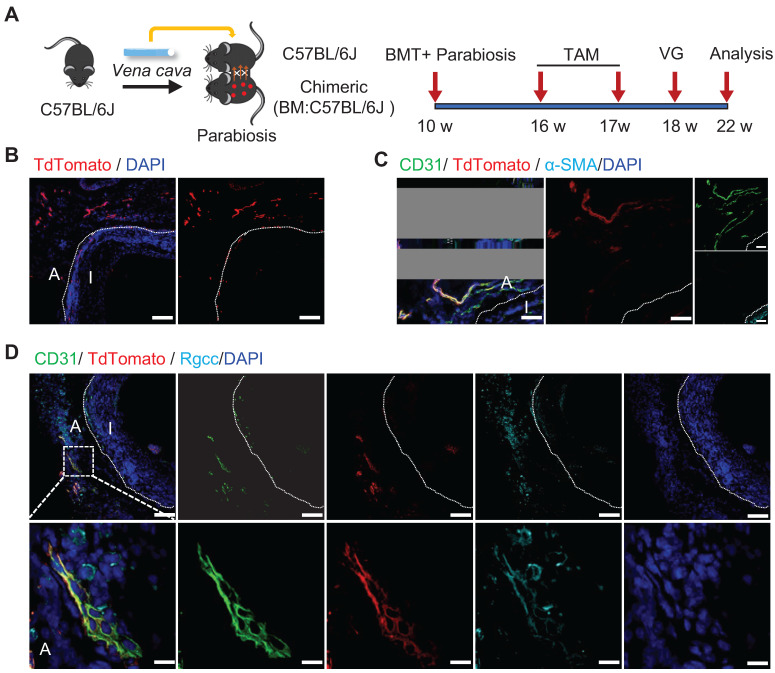
Non-bone Marrow Circulation Source of Sca-1+ Cells Forming Microvessels. (A) Strategy for parabiosis mouse model in which chimeric mice (bone marrow from C57BL/6J mice were transplanted to irradiated Ly6a-CreERT2; Rosa26-TdTomato mice) and C57BL/6J mice were subsequently sutured together. 6 weeks after after bone marrow transplantation and parabiosis, 5 pulses of tamoxifen were given to the mice. Subsequently, venae cavae from C57BL/6J mice were transplanted adjacent to the carotid arteries of parabiotic C57BL/6J mice. Grafts were collected 4 weeks after the surgeries. (B, C) Vein graft sections were stained with TdTomato, CD31 and α-SMA as indicated (Scale bars: 50 μm). Images shown are representative of n=6 separate grafts. (D) Vein graft sections were stained with TdTomato, CD31 and Rgcc as indicated (Scale bars: 50 μm, and 10 μm in enlarged image). Images shown are representative of n=6 separate grafts. Abbreviations: A, adventitia; α-SMA, α-Smooth Muscle Actin BMT, bone marrow transplantation; I, intima; Rgcc, Regulator of cell cycle; TAM, tamoxifen; VG, vein graft.

Non-bone-marrow-derived cells remained a mixed cell population, although we have demonstrated their important role in vein graft remodeling. Obviously, the non-bone-marrow-derived population in our study comprised resident cells of the carotid arteries and non-bone-marrow circulatory cells. To further examine the roles of specific sources of cells, we constructed a parabiosis mouse model in which the vessels from chimeric mice described above and C57BL/6J wild-type (WT) mice were sutured together (Figure 5A). In this model, both lines of mice shared a chimeric circulation. Therefore, the tdTomato^+^ cells detected in the vessel wall of the WT mice in the parabiosis mouse model were thought to originate from non-bone-marrow circulation 44, 45. Subsequently, the venae cavae from C57BL/6J WT mice were transplanted adjacent to the carotid arteries in the parabiotic WT mice (n = 6 experiments). Not surprisingly, no tdTomato^+^ cells were detected in the intima, as reported previously 9, 45. However, we observed abundant tdTomato^+^ cells in the adventitia (Figure 5B). Importantly, rather than expressing α-SMA (Figure 5C), some tdTomato^+^ cells expressed CD31 and RGCC (Figure 5D). These results indicated that non-bone-marrow-derived circulatory Sca-1^+^ cells mainly formed adventitial microvessels, but not a regenerated endothelium, in vein grafts. Accordingly, the Sca-1^+^ cells that generated ECs and SMCs in the intima/neointima of the vein grafts were thought to be derived from the recipient carotid arteries.

### Donor Sca-1^+^ Cells Mainly Dominated SMC Commitment in the Neointima at the Middle Bodies of Vein Grafts

**Figure 6:**
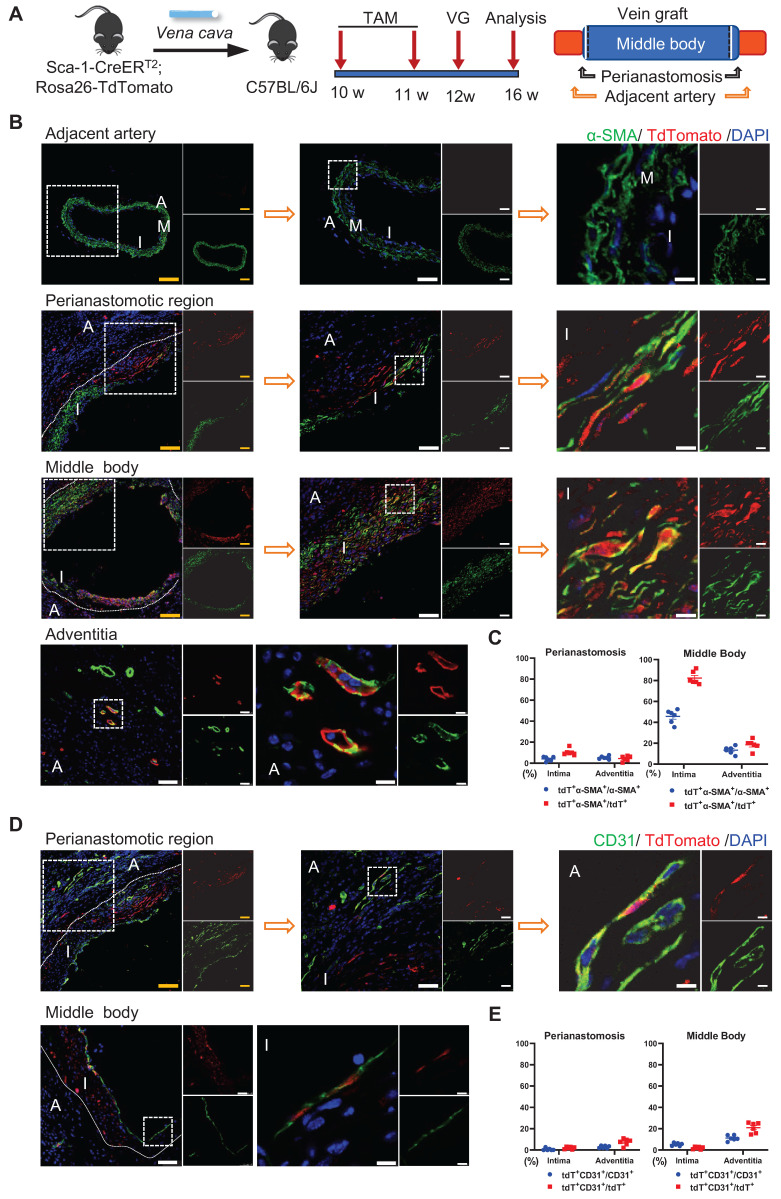
Venous Sca-1+ Cells Mainly Committing to SMCs at the Middle Bodies of Vein Grafts. (A) Schematic showing vein graft transplantation (n=6 experiments). After Ly6a-CreERT2; Rosa26-TdTomato mice received tamoxifen treatment, segments of venae cavae were transplanted adjacent to carotid arteries of C57BL/6J mice. The grafts were collected 4 weeks after the surgeries. (B) Vein graft sections at perianastomotic regions and middle bodies, along with adjacent carotid arteries, were stained with TdTomato and α-SMA as indicated (Scale bars: 100 μm in yellow, 50 μm in white, and 10 μm in enlarged image). Images shown are representative of n=6 separate grafts. (C) The panel representing percentages of cells expressing TdTomato in α-SMA+SMCs, or cells expressing α-SMA in TdTomato+ cells. All the data represent mean ± SEM, n=6 per group. (D) Vein graft sections at perianastomotic regions and middle bodies, along with adjacent carotid arteries, were stained with TdTomato and CD31 as indicated (Scale bars: 100 μm in yellow, 50 μm in white, and 10 μm in enlarged image). Images shown are representative of n=6 separate grafts. (E) The panel representing percentages of cells expressing TdTomato in CD31+ ECs, or cells expressing CD31 in TdTomato+ cells. All the data represent mean ± SEM, n=6 per group. Abbreviations: A, adventitia; α-SMA, α-Smooth Muscle Actin; I, intima; M, media; TAM, tamoxifen; VG, vein graft

The Sca-1^+^ cells from donor veins were the alternative sources for vein graft remodeling; therefore, we next assessed their roles using another vein graft model. In this mouse model, Sca-1-CreER^T2^; Rosa26-tdTomato mice were pulsed with tamoxifen, as shown in Figure 6A; the venae cavae were harvested 1 week after the administration of the last dose of tamoxifen and transplanted adjacent to the carotid arteries of the C57BL/6J WT mice (n = 6 experiments) (Figure 6A). Four weeks after the surgeries, a *de novo*-formed neointima combined with an expanded adventitia were observed at both the anastomotic regions and the middle bodies of the vein grafts (Figure 6B, 6D). More importantly, tdTomato^+^ cells were detected in both the neointima and the adventitia, indicating that Sca-1^+^ cells from the veins might be activated and participate in vein graft remodeling (Figure 6B, 6D). In both layers, some tdTomato^+^ cells expressed SMC markers, such as α-SMA, SM22, and Calponin, especially at the middle bodies (Figures 6B and S7A-B). Statistically, we found that, at the perianastomotic regions, only 4.03% ± 0.86% and 5.22% ± 0.63% of α-SMA^+^ SMCs were labeled by tdTomato in the neointima and the adventitia, respectively (Figure 6C). In contrast, at the middle bodies, tdTomato was expressed in 45.69% ± 2.65% and 13.39% ± 1.48% of α-SMA^+^ SMCs in the neointima and adventitia, respectively (Figure 6C).

Subsequently, we continued to investigate whether the Sca-1^+^ cells from donor veins give rise to ECs. Immunostaining showed that tdTomato^+^ cells from donor veins expressed the EC marker CD31 in the vein grafts (Figure 6D). The results showed that, at middle areas, 5.29% ± 0.55% and 11.19% ± 1.04% of CD31^+^ ECs displayed tdTomato labeling, possibly for endothelium remodeling and microvessel formation, respectively (Figure 6E). At perianastomotic regions, only 0.64% ± 0.55% and 2.85% ± 0.49% of CD31^+^ ECs displayed tdTomato in the intima and adventitia, respectively (Figure 6E). In addition, the tdTomato^+^ ECs also showed positive staining for other important EC markers, such as VE-cadherin and VWF (Figure S7C-D). Collectively, these data suggest that Sca-1^+^ cells from a venous source mainly contribute to neointima hyperplasia via SMC commitment, and, albeit with marginal significance, are involved in EC formation, especially at the middle areas of vein grafts.

### Knockdown of PDGFα in Sca-1^+^ Vascular Cells Decreased the Number of Intimal SMCs in Vein Grafts

**Figure 7:**
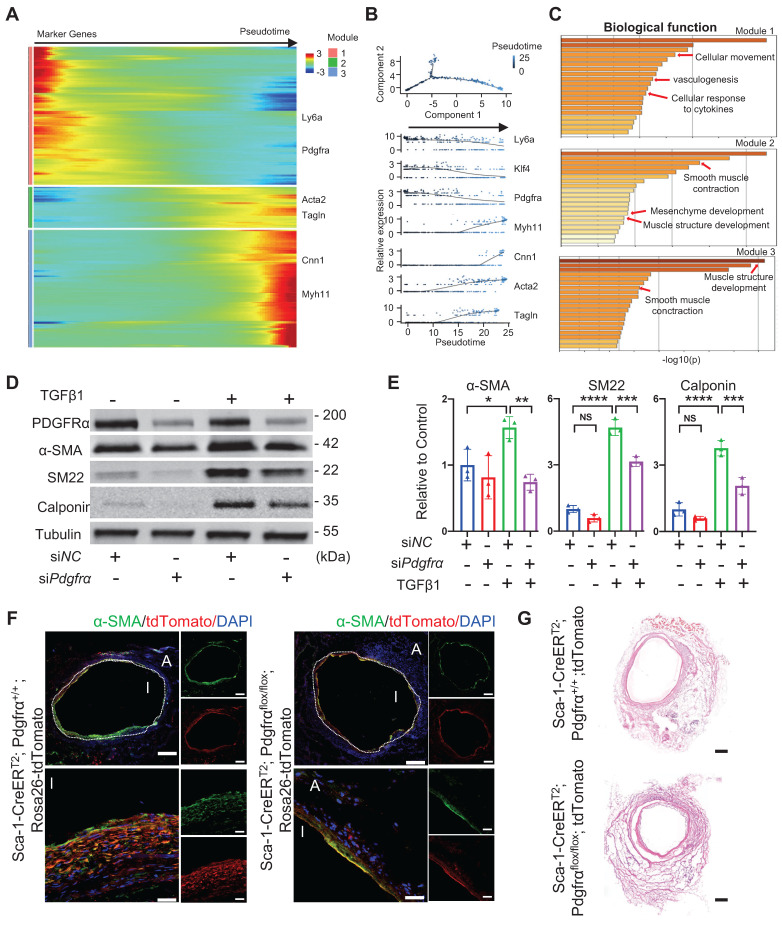
Analysis of Sca-1+ Cells to SMC Commitment in Vein Grafts (A, B) Heatmap showing pseudotime trajectory of mixing cells including smooth muscle cells and adventitial cells in sc-RNAseq of vein grafts. Expression of Ly6a, Klf4, Pdgfra, Myh11, Cnn1, Acta2 and Tagln were shown along the trajectory. (C) Biological function analysis of enrichment genes in 3 separated modules. (D, E) Western bolt and quantitative analysis showing protein expression of PDGFRα and SMC markers (SMA, SM22 and Calponin) in TGFβ1 treated Sca-1+ cells that were transfected with siNC or siPdgfrα. (F, G) Sca-1CreERT2; Pdgfrαflox/flox; Rosa26-tdTomato mice were given 5 pulses of tamoxifen. One week after the tamoxifen treatment, Vein grafts were performed in which both the donors and recipients were from Sca1CreERT2; Pdgfrαflox/flox; Rosa26-tdTomato mice. As a control, Sca-1CreERT2; Pdgfrα+/+ ; Rosa26-tdTomato mice were used in both donors and recipients in vein grafts. Grafts were collected 4 weeks after the surgeries. Vein graft sections at middle bodies were stained with TdTomato and α-SMA (F) and were performed with H&E staining (G) as indicated (Scale bars: 200 μm and 40 μm in enlarged image). Images shown are representative of n=4 separate grafts, respectively. Abbreviations: α-SMA, α-Smooth Muscle Actin; NC, Negative control; Pdgfrα, Platlet derived growth factor receptor α; SM22, Transgelin; sc-RNAseq, single cell RNA sequencing; TGFβ1, tissue growth factor β1.

Subsequently, to identify potential mechanisms underlying SMC commitment, all adventitial progenitors and SMCs annotated in the sc-RNA-seq experiment were pooled together for pseudotime trajectory analysis (Figure 7A). The pseudotime trajectory mimicked the process of SMC commitment, in which all genes were further assigned to three modules. Pseudotime-ordered cells clearly exhibited variable levels of specific markers in a trajectory manner, in which stem cell markers, including *Ly6a*, *Klf4*, and *Pdgfra*, were enriched in the head region, whereas mature SMC markers, including *Acta2*, *Tagln*, *Myh11*, and *Cnn1*, were located at the tail end (Figure 7A and 7B). The potential of Sca-1^+^ progenitors for SMC commitment was also confirmed in *in vitro* studies. We used microbeads to isolate adventitial Sca-1^+^ cells that exhibited low expression of SMC markers, as described previously 12. The results indicated that, in addition to progenitor cells, adventitial Sca-1^+^ cells may also include mature SMC-transformed stem-like cells that may still show low expression of contractile markers, as observed in our previous study 10, 20.

We used microbeads to isolate adventitial Sca-1^+^ cells (Figure S8A). After the Sca-1^+^ cells were treated with TGFβ1, they showed increased expression of contractile markers at both the gene and protein levels (Figure S8B-C). According to the trajectory, three modules were demonstrated based on their featured biological properties (Figure 7C). Module 1 mainly included genes related to cell movement, cytokine response, and vasculogenesis, whereas modules 2 and 3 were enriched in genes that were responsible for SMC differentiation and contraction (Figure 7C). The process of SMC commitment was possibly regulated by changes in transcription factors, such as *Klf4*, F*oxs1*, *Myocd*, and *Smad7*, as shown in the pseudotime trajectory (Figure S8D). In addition, we found that extracellular matrix genes, such as *Fn1*, *Fbn1*, *Col8a1*, *Col4a1*, *Lama5*, and *Acan*, were dynamically regulated during the process of SMC commitment (Figure S8E).

Because of the different fates of vascular Sca-1^+^ cells/progenitors and bone-marrow-derived Sca-1^+^ cells, as shown above, we performed bulk RNA-seq on the adventitial Sca-1^+^ cells and compared their gene expression with that observed in bone-marrow-derived Sca-1^+^c-Kit^+^Lin^-^ cells (LSKs). The bulk RNA-seq data of LSKs were downloaded from the public GSE216794 dataset. We found that the genes that were highly expressed in Sca-1^+^ vascular cells were related to vascular development, tube morphology, and extracellular matrix organization, whereas those of LSKs were mainly related to the inflammatory response and leucocyte activation (Figure S8F-G). Based on the data obtained from both sc-RNAseq and bulk RNA-seq, we found that Pdgfrα was highly expressed in Sca-1^+^ vascular cells; therefore, subsequently, we assessed the function of Pdgfrα in Sca-1^+^ vascular cells. First, we transfected Sca-1^+^ vascular cells with si*NC* or si*Pdgfrα.* Then, the cells were treated without (as a negative control) or with TGFβ1. Our western blotting results showed that, after knockdown of Pdgfrα, the expression of SMC markers in Sca-1^+^ vascular cells that were under the stimulation of TGFβ1 was reduced (Figure 7D-E). Next, by crossing Pdgfrα^flox/flox^, Sca-1-CreER^T2^, and Rosa26-tdTomato reporter mice, we constructed Sca-1(Ly6a)-CreER^T2^; Pdgfrα^flox/flox^; Rosa26-tdTomato mice. After genotyping, the Pdgfrα^flox/flox^; Sca-1-CreER^T2^; Rosa26-tdTomato mice were administered five pulses of tamoxifen. One week later, the venae cavae from the Pdgfrα^flox/flox^; Sca-1-CreER^T2^; Rosa26-tdTomato mice were harvested and transplanted adjacent to the carotid arteries of other tamoxifen-pulsed Pdgfrα^flox/flox^; Sca-1-CreER^T2^; Rosa26-tdTomato mice (n = 4 experiments). Thus, in this mouse model, both the recipients and the donors were Pdgfrα^flox/flox^; Sca-1-CreER^T2^; Rosa26-tdTomato mice. Four weeks later, the vein grafts were collected for further analysis. The Pdgfrα^+/+^; Sca-1-CreER^T2^; Rosa26-tdTomato mice were used as a control (n = 4 experiments), and the procedures used, which included tamoxifen treatment, vein graft transplantation, and tissue analysis, were similar to those employed to analyze the Pdgfrα^flox/flox^; Sca-1-CreER^T2^; Rosa26-tdTomato mice. The results showed that conditional knockout of Pdgfrα in Sca-1^+^ cells decreased the number of SMCs in the intima of vein grafts *in vivo* (Figure 7F-G)*.* Collectively, these results provided the potential mechanisms underlying the transformation of Sca-1^+^ vascular cells/progenitors into SMCs, suggesting that knockdown/knockout of Pdgfrα in Sca-1^+^ cells is a strategy that may be employed to postpone vein graft failure.

## Discussion

In the current study, we utilized several techniques (including sc-RNAseq, lineage-tracing, vein graft and/or bone-marrow transplantation, and parabiosis mouse models) to identify the cell sources and their functions in vein grafts. Using sc-RNAseq, we first confirmed that diverse sources of Sca-1^+^ cells may mutually participate in vascular remodeling. Subsequently, using the genetic-lineage-tracing technique, we provided evidence that recipient arteries, donor veins, non-bone-marrow circulation, and the bone marrow provided diverse Sca-1+ progenitors that function in variable manners in the reshaping of vein grafts.

Reendothelialization is vital for preserving vascular function. Heterogenous sources are reported to generate ECs after vascular injury 46. The circulation has been proposed to harbor endothelial progenitor cells that engraft into the endothelium and are responsible for EC restoration 8, 42, 43. Resident ECs constitute another source of cells that can regenerate the impaired endothelium 9, 45. In line with these studies, our sc-RNAseq data suggested that several lines of cells restore the endothelium in vein grafts. Statistically, the results obtained from several mouse models demonstrated that, at the perianastomotic regions, 74.50% ± 3.33% of ECs were derived from recipient/arterial Sca-1^+^ cells, whereas only 0.64% ± 0.55% of those cells stemmed from the venae cavea of the donors. In contrast, at the middle bodies, only 3.45% ± 1.03% and 5.29% ± 0.55% of ECs were derived from the arterial or venous Sca-1^+^ cells, respectively. In addition, no circulatory Sca-1^+^ cells were involved in endothelium regeneration. Of note, although a minor population of venous ECs expressed Sca-1 in our study, we were unable to determine whether these cells were derived from expanded Sca-1^+^ progenitors or damaged ECs that temporarily expressed the Sca-1 marker. Wu et al. 10 reported a similar result in that flanking recipient ECs regenerated the endothelium, especially at the perianastomotic regions of vein grafts; however, they did not address the exact cell population involved in this process. Our study provided evidence that local Sca-1^+^ cells from an arterial source dominated luminal restoration, particularly at the perianastomotic regions. Moreover, because of their remarkable capability for expansion, they are probably EC progenitors, as proposed by Naito et al. 15 and Tang et al. 19. However, because of the heterogeneity of ECs, the results of our study did not exclude the existence of other cell populations that restore the endothelium of vein grafts. In fact, several progenitors expressing variable markers, including CD157 47, Procr 48, c-Kit 49, CD34 29, and Atf3, have been reported to contribute to endothelium restoration 45. Therefore, whether these cells participate in endothelium restoration warrants further examination. In summary, the results of our study suggested that resident progenitors from a recipient/arterial source are potential candidate cells for reendothelization and may serve as a clinical target to reduce the stenosis of vein grafts.

Our study also identified cell sources for the formation of adventitial microvessels in vein grafts. We found that, at the perianastomotic regions, 24.70% ± 2.08% of adventitial microvessels were derived from the recipient mice, whereas only 2.85% ± 0.49% of adventitial microvessels were from the donor veins; at the middle bodies, the recipients and the donors contributed with 12.70% ± 0.96% and 11.19% ± 1.04% of the adventitial microvessels of vein grafts, respectively. Notably, although circulatory Sca-1^+^ cells do not regenerate the endothelium, we identified a population of non-bone-marrow-derived Sca-1^+^ cells that formed adventitial microvessels. To the best of our knowledge, this was the first evidence based on lineage-tracing mouse models to show that circulatory cells participate in EC formation, although the functions of these microvessels warrant further investigation. It is widely accepted that microvessels in both the intima and the adventitia can accelerate the development of atherosclerosis 50; conversely, the microvessels also supply nutrients and oxygen to the vessel wall, which probably affords a protective effect. Furthermore, the microvessels in vein grafts may also play a role in stabilizing them and accelerating arterialization by recruiting mesenchymal cells, such as SMCs, pericytes, and/or other vascular progenitors. This is evidenced by our finding that Sca-1^+^ cells from recipient mice formed 60.33% ± 3.48% of the adventitial SMCs detected at the perianastomotic regions of vein grafts, whereas Sca-1^+^ cells from donor veins contributed to 13.39% ± 1.48% of the adventitial SMCs observed at the middle bodies of vein grafts. In our study, donor veins, recipient carotid arteries, and non-bone-marrow circulation all provided Sca-1^+^ cells for microvessel formation. In agreement with previous studies 29, 49, Sca-1^+^ cells derived from the bone marrow were not involved in microvessel formation. Moreover, the results of our study broadened the pathophysiological knowledge of vein grafts by providing an alternative cellular source for vein graft remodeling. Further studies may strive to detect the function of these non-bone-marrow-derived circulatory progenitors. For example, future studies may be designed to detect the effects of the toxins (such as urea) that are commonly observed in chronic kidney diseases on these newly identified cells, and, more importantly, to translate the results into clinical settings. In terms of the exact sources of these cells, we propose that, in response to mediators, progenitors resident in other organs, such as the liver, lung, and kidney, may egress, form a mixed cell pool, migrate into vein grafts, and finally form adventitial microvessels. In addition, whether the function and/or number of these cells are independent indexes to predict the outcomes of vein grafts can be tested in further clinical studies.

Based on a substantial number of studies, several cell sources have been proposed to form SMCs in the neointima in different cardiovascular disease models. Medial SMCs were reported as a main cause of intimal hyperplasia in various lineage-tracing systems 10, 51-56; however, the conclusions of those studies did not rule out the contribution of other cell sources. Recently, using severe vascular injury models, including allograft transplantation and arterial anastomosis, our group 57 and Tang et al. 18 demonstrated that progenitor cells are also prominent sources for the generation of SMCs in the neointima. Similar results were also described by Kramann et al. 58, who showed that Gli1^+^ progenitors migrate from the adventitia into the neointima and differentiate into SMCs in severe renal diseases. Other than the vessel wall, the circulation was also shown to harbor SMCs 40, 41. Our study demonstrated that the Sca-1^+^ cells from recipient arteries and donor veins formed 13.43% ± 2.06% of the neointimal SMCs at the perianastomotic regions and 45.69% ± % 2.65% of the neointimal SMCs at the middle bodies, respectively. In contrast, no circulating Sca-1^+^ cells (including those from bone-marrow and non-bone-marrow sources) contributed to the accumulation of neointimal SMCs. These results suggest that Sca-1^+^ cells from the donor veins and recipient carotid arteries migrate into the neointima and form SMCs in vein grafts. However, the exact source of these Sca-1^+^ cells remains elusive. Our transcriptomics results indicated that Sca-1^+^ cells that formed SMCs were probably adventitial progenitors, which was consistent with the conclusion drawn by Tang et al. 18. In turn, Dobnikar et al. 14 showed that Sca-1 is also expressed in some media SMCs, which were suggested as another line of progenitors that accelerate neointimal hyperplasia. Recently, Wu et al. 10 and Majesky et al. 20 clearly showed that a proportion of mature SMCs express Sca-1 and transform into stem-like cells during vascular remodeling. In addition, Chen et al. showed that 3%-7% of SMCs in the neointima of vein grafts were derived from arterial ECs via the endothelial-to-mesenchymal transition 59. These cells may play a role, albeit marginal, in SMC differentiation in vein grafts. Therefore, we propose that Sca-1^+^ cells/progenitors from these sources may comprise a mixed cell pool and transform into SMCs in response to altered blood flow and the cytokines released in vein grafts. Moreover, our study also provided evidence that knockdown/knockout of Pdgfrα in Sca-1^+^ cells decreased the cell potential to generate SMCs *in vitro* and decreased the number of intimal SMCs in vein grafts. However, we were unable to determine whether the remodeling shown in our study was a result of vein graft adaptation or pathological processes. Therefore, further studies are necessary to confirm the effects of the conditional knockout of *Pdgfrα* in Sca-1^+^ ECs on EndMT, which is thought to be a hallmark of the adaptive response 10.

The findings reported here provide an important pathophysiological understanding of, and have therapeutic implications for vein grafts. However, our study also had several limitations. First, it is widely known that Sca-1(Ly6a) is a cell-surface marker only in the mouse (and not in humans) 11. Therefore, further studies are necessary to illustrate the functions of the counterpart cells that express makers such as CD34, c-Kit, and Gli1 in human vein grafts 12, 13. Although less elucidated, CD34^+^ cells in the human vessel wall display progenitor characters that are similar to those of as Sca-1^+^ cells in the mouse 18, 29, 60-63. Therefore, we propose that, in humans, CD34^+^ cells may be a counterpart cell population of Sca-1^+^ cells. In addition, because of the heterogeneity and complexity of progenitor cells, human cells expressing different progenitor markers are other potential candidates to exert a similar function to that of Sca-1^+^ cells in the mouse; however, additional studies are necessary to illustrate this issue. Second, Wu et al. reported that, according to the neointimal hyperplasia and the lumen patency indexes, the remodeling of vein grafts was similar at 3 weeks after surgery, whereas they could be divided into severe (VG-S) or mild (VG-M) types at 6 weeks post-surgery 10. The VG-S and VG-M types of remodeling probably result in late vein graft failure and benign vein graft adaptation, respectively. In our vein graft mouse models, we collected the grafts at 4 weeks after the surgeries, when the vessel wall thickness and the neointimal area were remarkably increased, to obtain a vein graft remodeling that resembled the early adaptation of transplanted human saphenous veins for clinical bypass surgical treatment. However, further studies are necessary to examine the role of vascular progenitor cells in the intermediate-to-late stages of vein graft remodeling. Third, some tdTomato^+^ cells derived from either arterial or venous sources did not express EC or SMC markers in the adventitia of vein grafts. These cells might be a mixed cell population that includes undifferentiated adventitial progenitors and mature SMC-transformed stem-like cells. Therefore, further studies need to be performed to elucidate their functions.

In summary, our study provided an unbiased cell atlas of vein grafts. We demonstrated that, in vein grafts, the resident progenitors from donor veins mainly formed the neointimal SMCs at the middle bodies, whereas recipient progenitors from carotid arteries were mainly responsible for endothelium restoration at the perianastomotic regions. Based on these findings, further studies can be designed and performed on counterpart cells in human vessels, as mentioned above, to filter out the cell source that underlies the maintenance of graft patency by increasing endothelium restoration and decreasing the neointimal area. Furthermore, we demonstrated the existence of non-bone-marrow-derived circulating progenitors as a new cell source, and illustrated their vital role in the formation of the adventitial microvessels for vein graft remodeling. Future clinical studies can test whether the function and/or number of these cells are independent indexes that can be used to predict the outcomes of vein grafts.

## Supplementary Material

Supplementary figures.

## Abbreviations

α-SMA: α-smooth muscle actin
DAPI: 4',6-Diamidino-2-Phenylindole
DTA: diphtheria toxin
EC: endothelial cell
KNN: K-nearest neighbor
Ly6a: lymphocyte antigen 6 complex, locus A
PCA: principle component analysis
PCR: polymerase chain reaction
Pdgfrα: platelet-derived growth factor receptor α
Sca-1: stem cells antigen-1
scRNA-seq: single-cell RNA sequencing
SM22: transgelin
siRNA: small interfering ribonucleic acid
SMC: smooth muscle cell
TGFβ: transforming growth factor β
UAMP: uniform manifold approximation and projection
VE-Cadherin: vascular endothelial cadherin
VWF: von Willebrand factor
