# Supplementary material for: Multilineage commitment of Sca-1+ cells in reshaping vein grafts
Source: Theranostics. 2023 Apr 1;13(7):2154–75. doi: 10.7150/thno.77735 (PMC10157743; doi:10.7150/thno.77735)
Supplement: Supplementary file 1 — Supplementary figures. [file thnov13p2154s1.pdf]

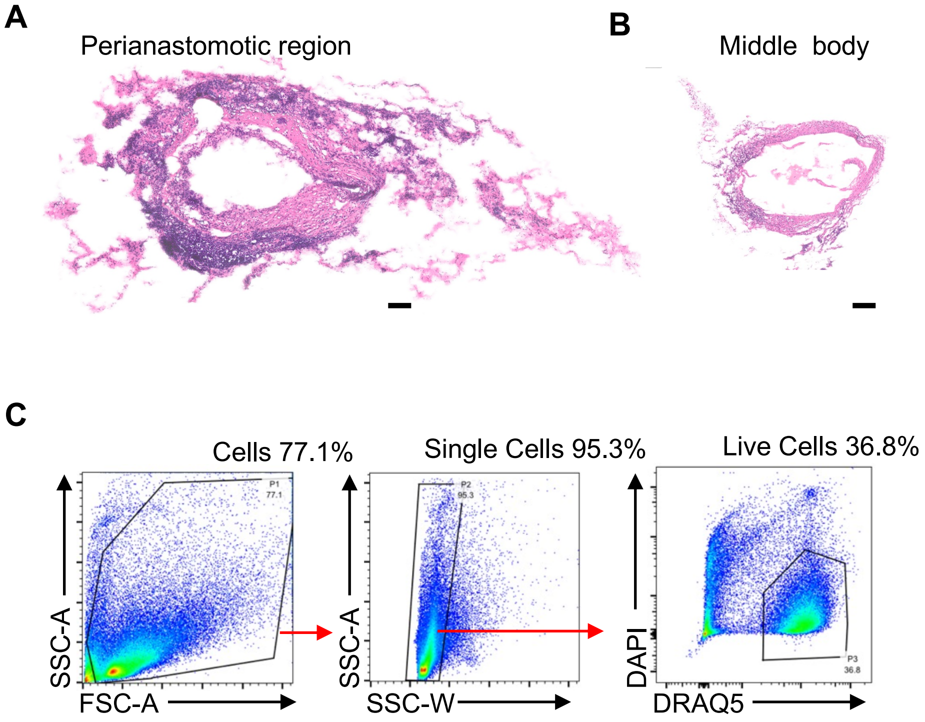

Figure S1. Tissue Characterization, Preparation and Analysis of Sc-RNAseq.
(A, B) The vena cava was grafted into the carotid artery. Representative H&E images showing perianastomotic regions and middle bodies within grafts that were harvested 4-weeks after vein graft (Scale bars: 200 µm).
(C) Representative gating strategy for sc-RNAseq.

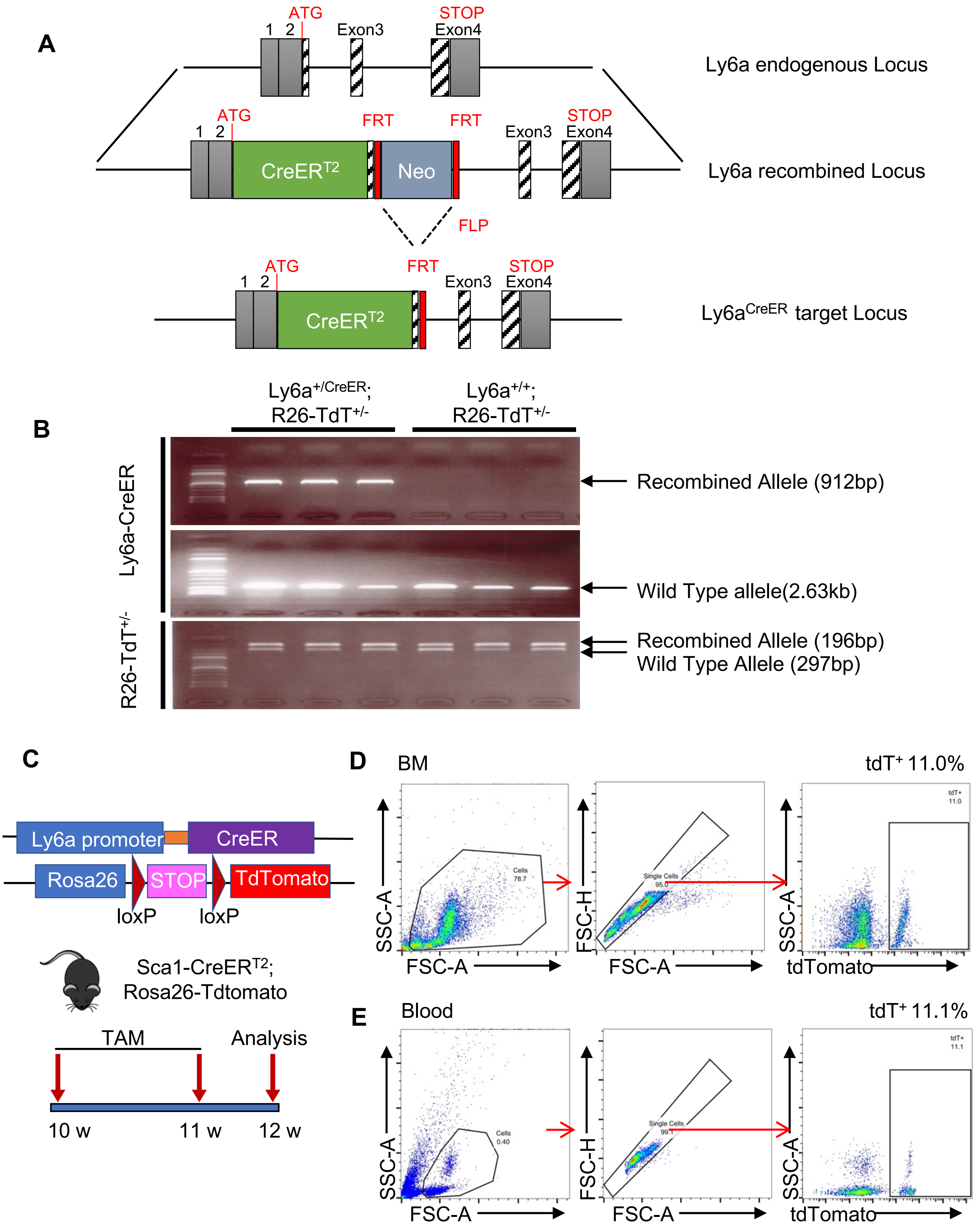

Figure S2. Ly6a-CreER^{T2}; Rosa26-tdTomato Mice Strategy and Identification.

(A) Strategy for Ly6a-CreER^{T2} allele generation.

(B) Conventional PCR showing genotyping for Ly6a-CreER^{T2}; Rosa26-tdTomato mice.

(C) Experimental graph showing Ly6a-CreER^{T2} mice that were crossed with Rosa26-TdTomato reporter mice line; strategy for experimental schedule of tamoxifen-induced TdTomato labelling of Sca-1^{+} cells

(D, E) Representative flow cytometric analysis of TdTomato^{+} cells in bone marrow and blood(n=6).

Abbreviations: BM, bone marrow; R26, Rosa-26;TdT, tdTomato.

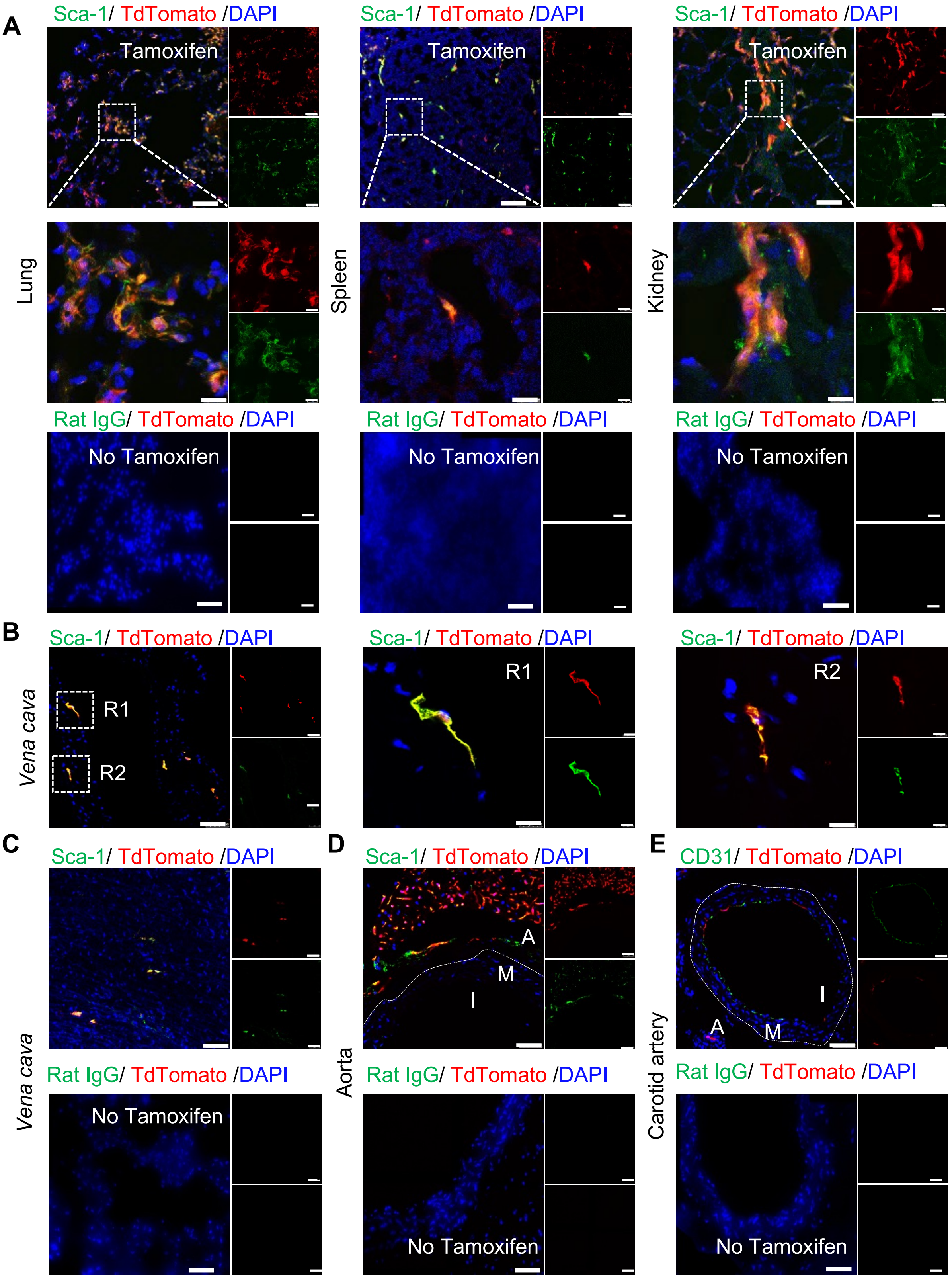

Figure S3. Verification of TdTomato Labeling in Ly6a-CreER^{T2}; Rosa26-tdTomato Mice.
(A) Ly6a-CreER^{T2}; Rosa26-tdTomato mice were treated with tamoxifen or no tamoxifen. Immunostaining showing TdTomato^{+} labelling of sca-1^{+} cells in lung, spleen and kidney (Scale bars: 50 μm, and 10 μm in enlarged image). Images shown are representative of n=3 separate mice.
(B) Immunostaining showing longitude section of vena cava from Ly6a-CreER^{T2}; Rosa26-tdTomato mice stained with TdTomato and Sca-1 (Scale bars: 50 μm, and 10 μm in enlarged image). Images shown are representative of n=3 separate vessels.
(C) En face staining of venae cavae showing TdTomato^{+} Sca-1^{+} cells (Scale bars: 50 μm). Images shown are representative of n=3 separate vessels.
(D, E) Immunostaining showing TdTomato^{+} cells in cross sections of aortas and carotid arteries from Ly6a-CreER^{T2}; Rosa26- TdTomato mice that were co-stained with Sca-1 and CD31, respectively (Scale bars: 50 μm). Images shown are representative of n=3 separate vessels.
Abbreviations: A, adventitia; I, intima; M, media; R1-2 indicates region 1-2.

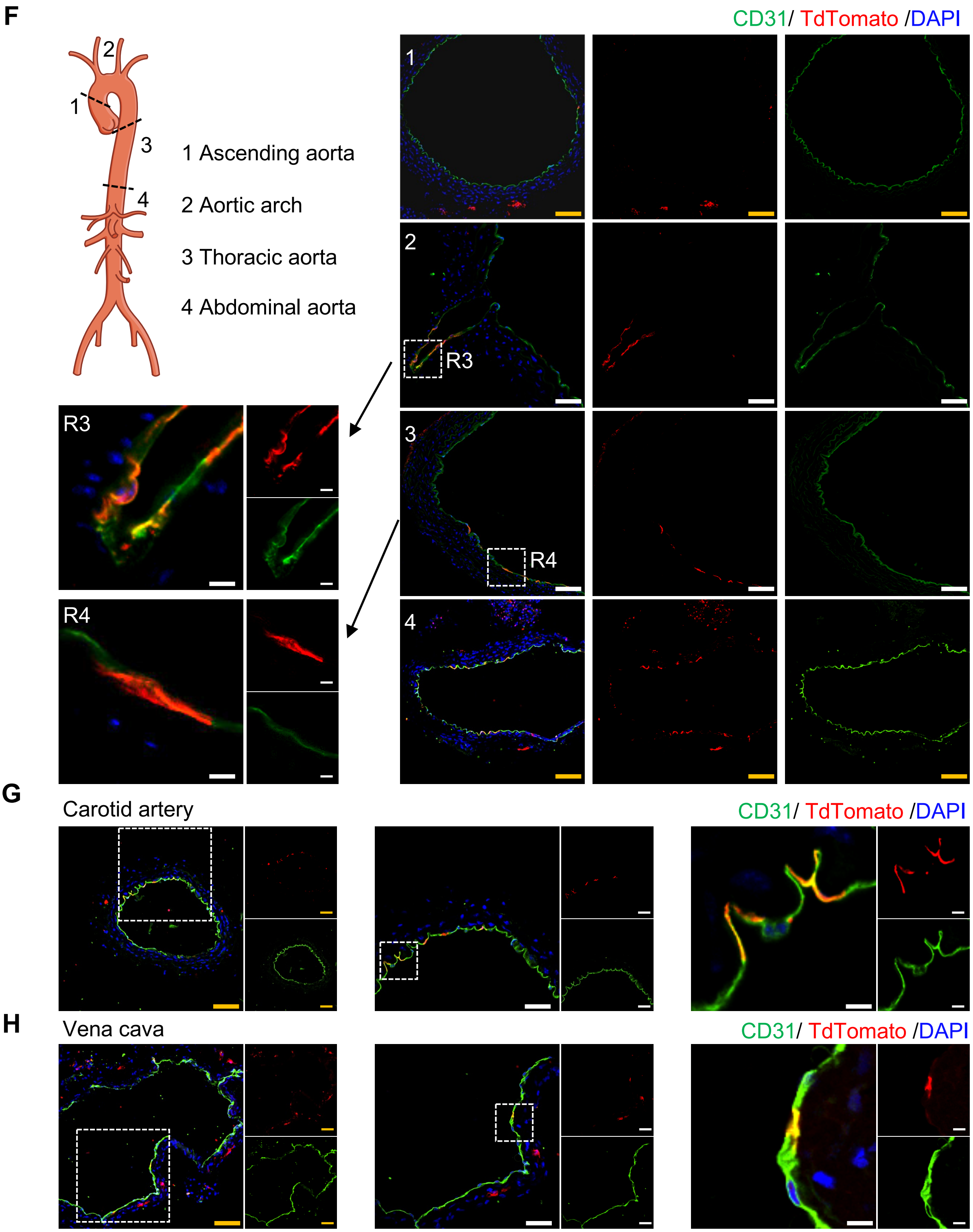

Figure S3. Verification of Sca-1$^+$ Cells in Aortas, Carotid Arteries and Venae Cavae

(F) Ly6a-CreER^{T2}; Rosa26-tdTomato mice were treated with tamoxifen. Aortas were divided into four segments for analysis. Representative immunostaining shows TdTomato$^+$ and CD31 labeling in aortas (Scale bars: 100 μm in yellow, 50 μm in white, and 10 μm in enlarged image). Images shown are representative of n=3 separate aortas.

(G) Immunostaining showing cross sections of carotid arteries from the Ly6a-CreER^{T2}; Rosa26-tdTomato mice stained with TdTomato and CD31 (Scale bars: 100 μm in yellow, 50 μm in white, and 10 μm in enlarged image). Images shown are representative of n=3 separate carotid arteries.

(H) Immunostaining showing cross sections of venae cavae from the Ly6a-CreER^{T2}; Rosa26-tdTomato mice stained with TdTomato and CD31 (Scale bars: 100 μm in yellow, 50 μm in white, and 10 μm in enlarged image). Images shown are representative of n=3 separate venae cavae.

Abbreviations: R3-4, region 3-4.

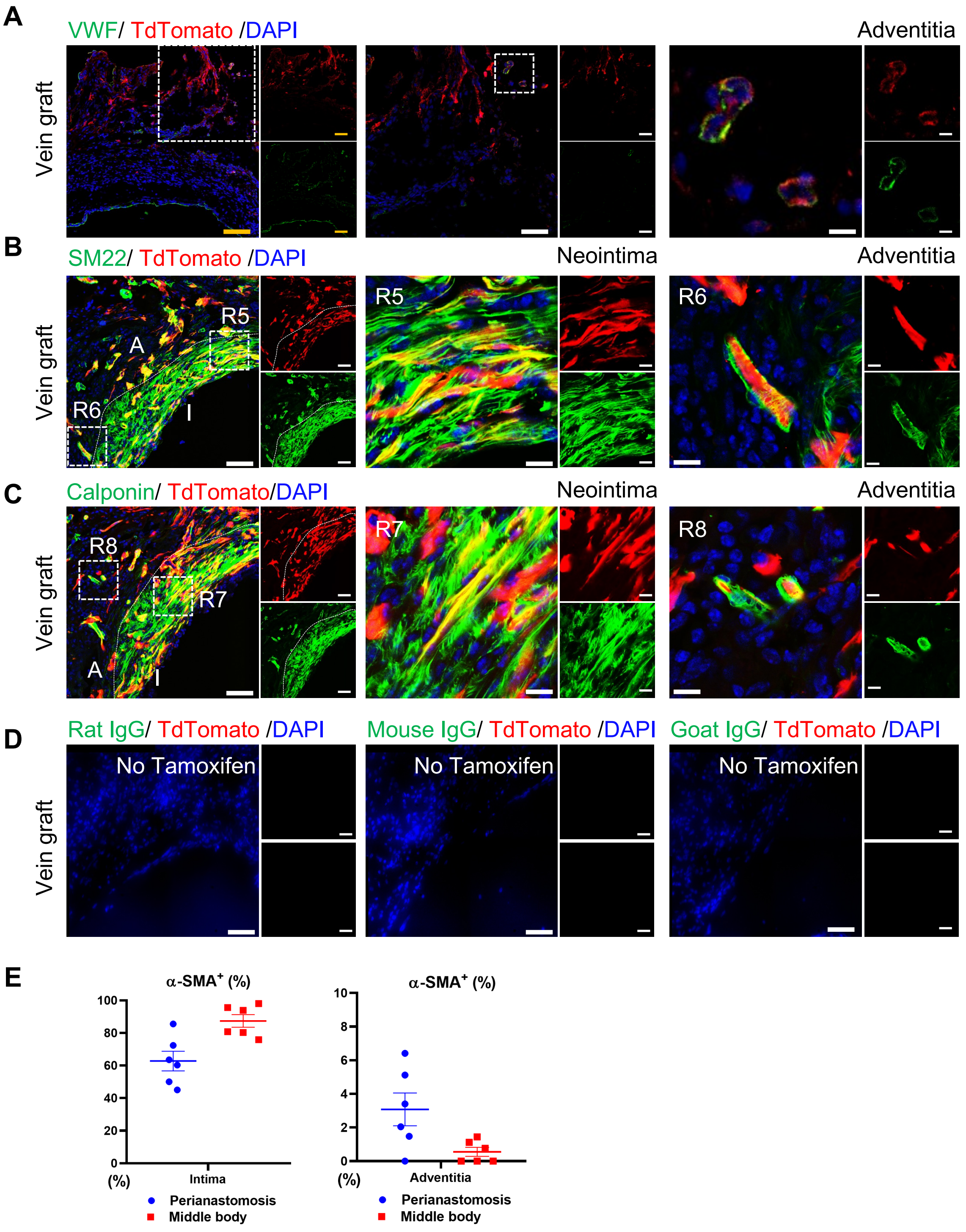

Figure S4. Recipient Sca-1⁺ Cells Generating Both ECs and SMCs in Vein Grafts.

(A) Vein graft sections were stained with TdTomato and VWF as indicated (Scale bars: 100 μm in yellow, 50 μm in white, and 10 μm in enlarged image). Images shown are representative of n=6 separate grafts.

(B and C) Vein graft sections were stained with TdTomato ,SM22 and Calponin as indicated (Scale bars: 50 μm, and 10 μm in enlarged image). Images shown are representative of n=6 separate grafts.

(D) Immunostaining showing cross sections of vein graft from Ly6a-CreER^{T2}; Rosa26-tdTomato without tamoxifen treatment stained with respective IgG control antibody (Scale bars: 50 μm).

(E) The panel showing percentage of α-SMA⁺ cells in the neointima and the adventitia within the perianastomotic regions and middle bodies of vein grafts. All the data represent mean ± SEM, n=6 per group.

Abbreviations: A, adventitia; I, neointima; R1-8, region 1-8; SM22, Transgelin.

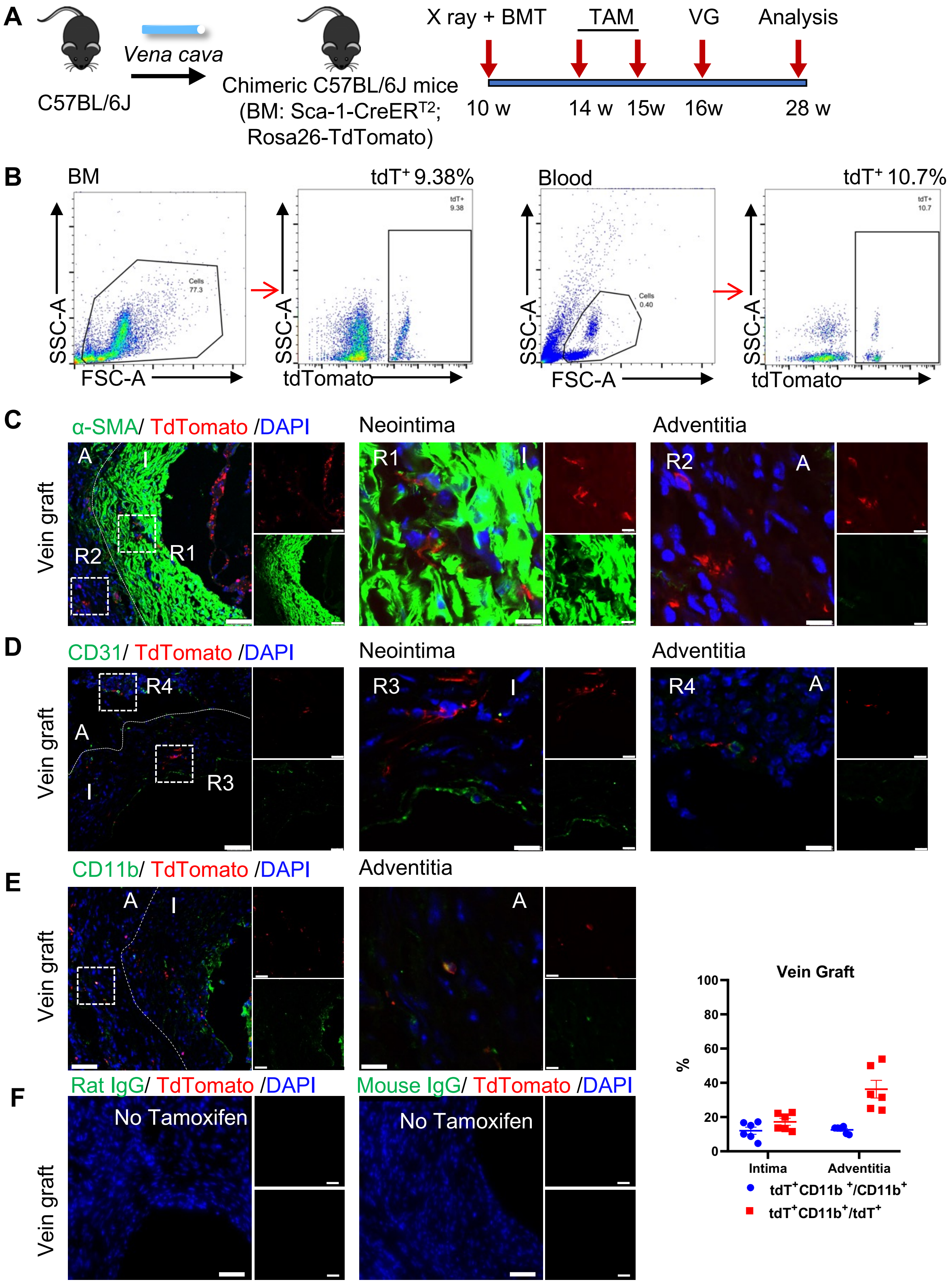

Figure S5. Bone Marrow Source of Recipient Sca-1^{+} cells Differentiating into Inflammatory Cells.

(A) Strategy for chimeric mouse model in which bone marrow cells from Ly6a-CreER^{T2}; Rosa26-TdTomato mice were transplanted to irradiated C57BL/6J mice. 4 weeks after bone marrow transplantation, pulses of tamoxifen were given to the chimeric mice. Subsequently, venae cavae from C57BL/6J mice were transplanted adjacent to the carotid arteries of the chimeric mice. Grafts were collected 4 weeks after the surgeries.

(B) Representative flow cytometric analysis of TdTomato^{+} cells from bone marrow and blood in chimeric mice(n=6).

(C, D) Vein graft sections were stained with TdTomato, α-SMA and CD31 as indicated (Scale bars: 50 μm, and 10 μm in enlarged image). Images shown are representative of n=6 separate grafts.

(E) Vein graft sections were stained with TdTomato and CD11b as indicated (Scale bars: 50 μm, and 10 μm in enlarged image). Images shown are representative of n=6 separate vessels or grafts. The panel representing quantification percentage of TdTomato expression in CD11b^{+} cells or CD11b expression in TdTomato^{+} cells. All the data represent mean ± SEM, n=6 per group.

(F) Immunostaining showing cross section of vein graft stained with IgG control antibody (Scale bars: 50 μm).

Abbreviations: A, adventitia; α-SMA, α-Smooth Muscle Actin; I, neointima; R1-4, region 1-4; TdT, TdTomato.

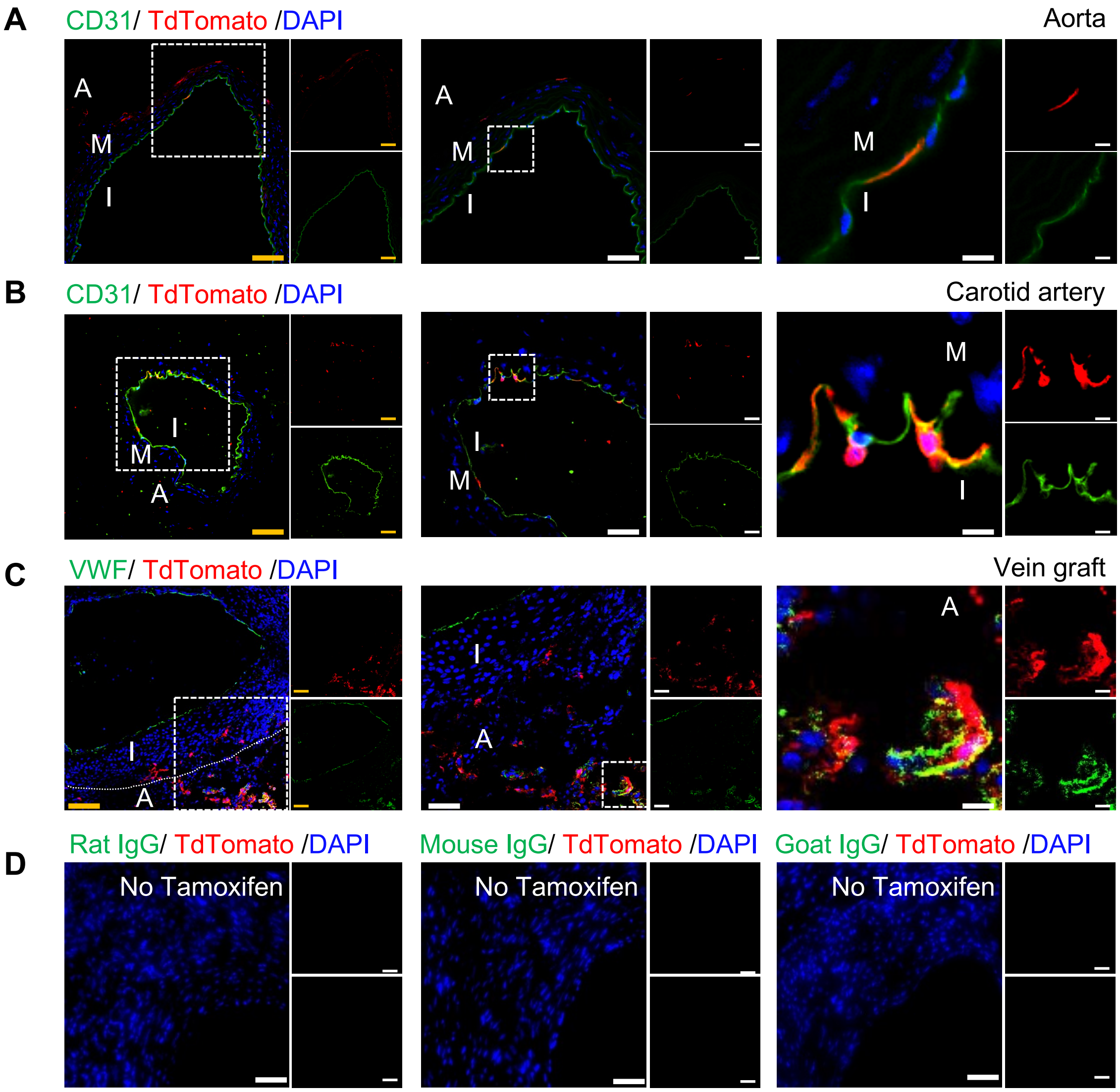

Figure S6. Non-bone Marrow Source of Sca-1⁺ Cells in Vein Grafts.
(A) Chimeric mice were produced in which bone marrow from C57BL/6J mice were transplanted to irradiated Ly6a-CreER^{T2}; Rosa26-TdTomato mice. Immunostaining showing cross sections of aortas from the chimeric mice that are stained with TdTomato and CD31 (Scale bars: 100 µm in yellow, 50 µm in white, and 10 µm in enlarged image). Images shown are representative of n=6 separate vessels.
(B) Immunostaining showing cross sections of carotid arteries from the chimeric mice that are stained with TdTomato and CD31 (Scale bars: 100 µm in yellow, 50 µm in white, and 10 µm in enlarged image). Images shown are representative of n=6 separate vessels.
(C) Four weeks after bone marrow transplantation, pluses of tamoxifen were given to the chimeric mice. Subsequently, venae cavae from C57BL/6J mice were transplanted adjacent to the carotid arteries of the chimeric mice. Grafts were collected 4 weeks after the surgeries . Representative of immunostainings showing vein graft sections that were stained with TdTomato and VWF as indicated (Scale bars: 100 µm in yellow, 50 µm in white, and 10 µm in enlarged image). Images shown are representative of n=6 separate grafts.
(D) Immunostaining showing cross sections of vein grafts from Ly6a-CreER^{T2}; Rosa26- TdTomato mice without tamoxifen treatment stained with respective IgG control antibody (Scale bars: 50 µm).
Abbreviations: A, adventitia; I, intima; VWF, Von Willebrand Factor.

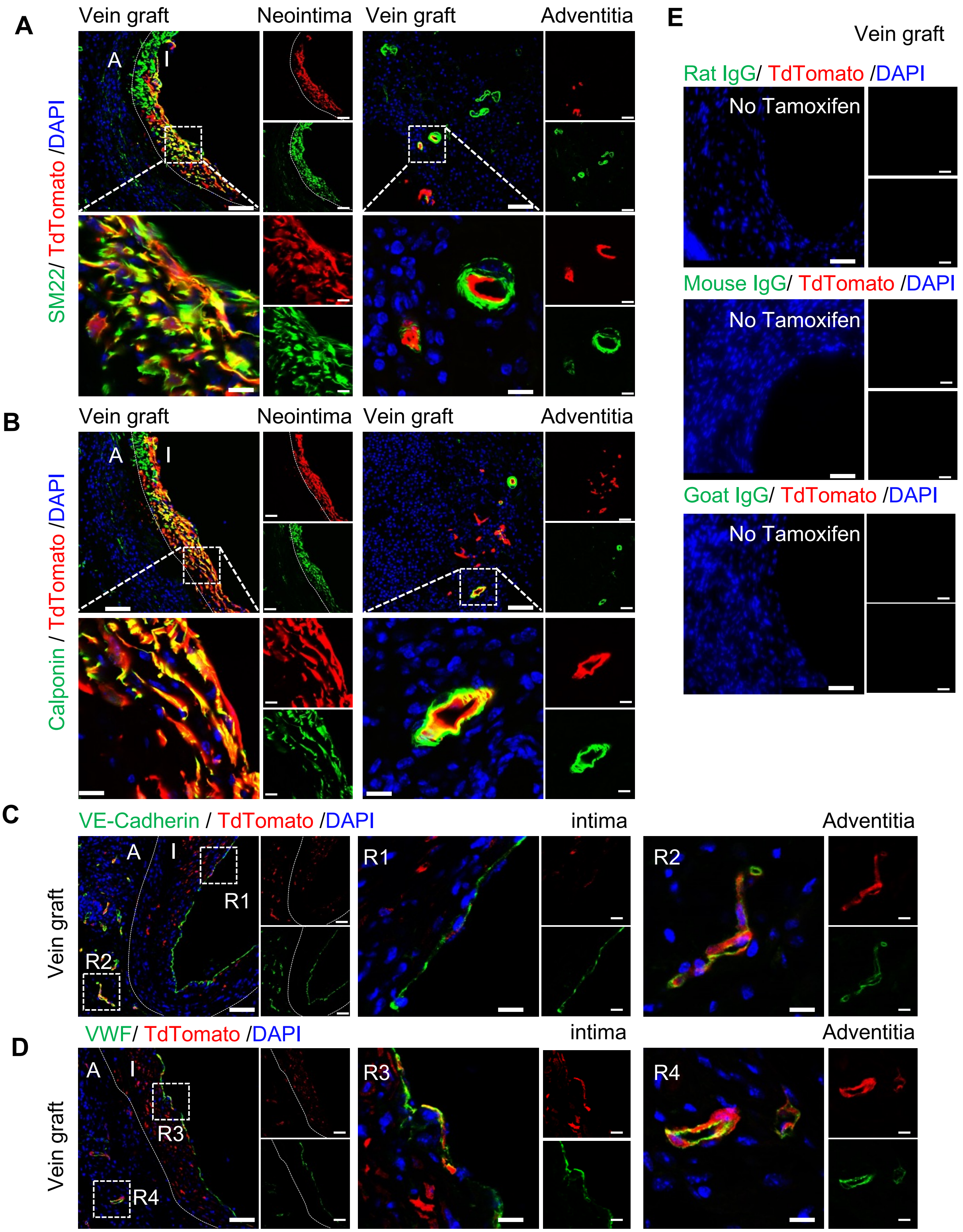

Figure S7. Venous Sca-1⁺ Cells Mainly Giving Rise to SMCs in the Neointima.
(A, B) Vein graft sections were stained with TdTomato, SM22 and Calponin as indicated (Scale bars: 50 µm, and 10 µm in enlarged image). Images shown are representative of n=6 separate grafts.
(C, D) Vein graft sections were stained with TdTomato, CD144 and VWF as indicated (Scale bars: 50 µm, and 10 µm in enlarged image). Images shown are representative of n=6 separate grafts.
(E) Immunostaining showing cross section of vein graft from Ly6a-CreER^{T2} ; Rosa26- TdTomato mice without tamoxifen treatment stained with respective IgG control antibody (Scale bars: 50 µm).
Abbreviations: A, adventitia; I, intima; R1-4, region 1-4; SM22, Transgelin; VWF, Von Willebrand Factor.

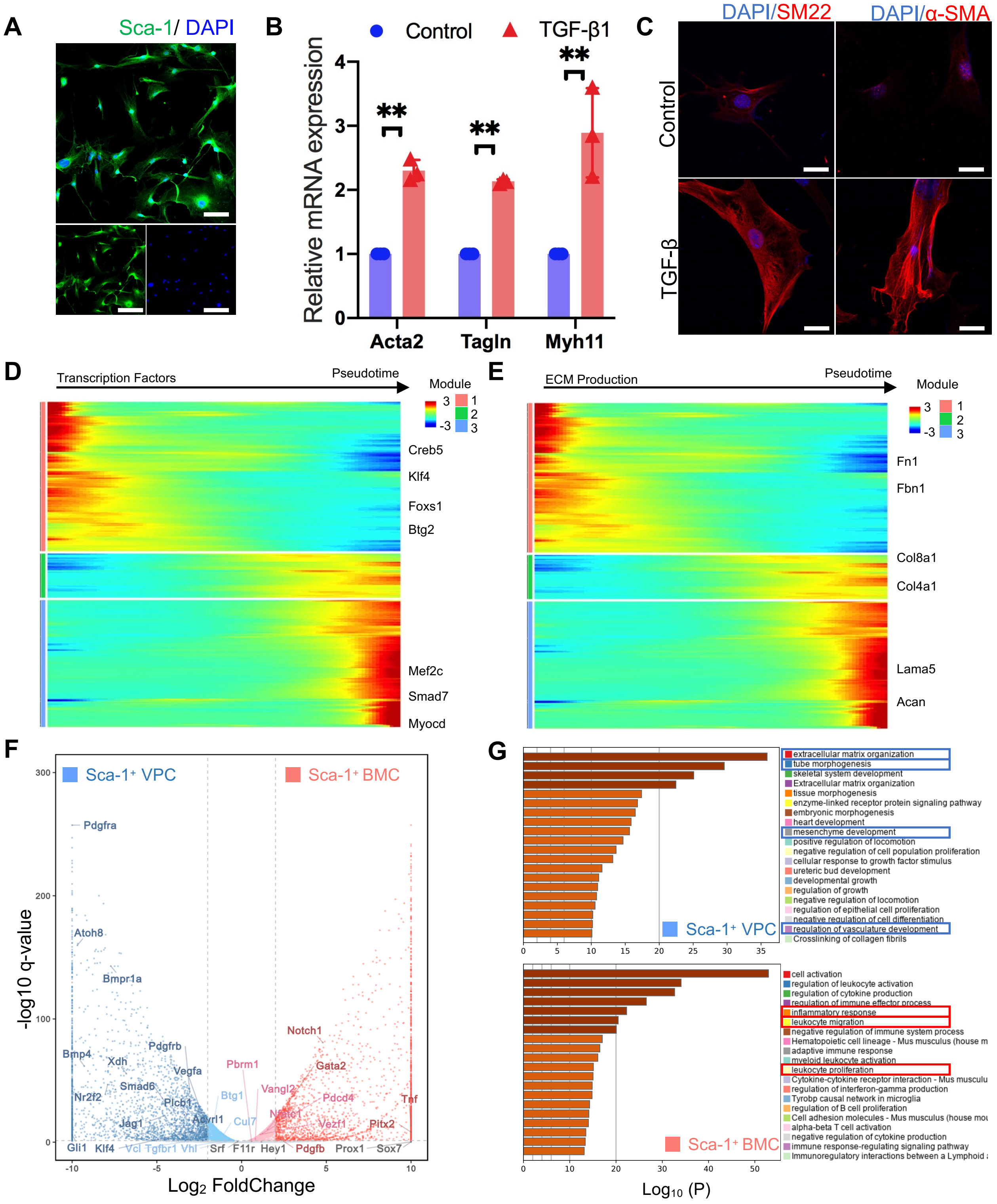

Figure S8. Gene Expression of Sca-1$^+$ Cells in Vessel Wall and Bone Marrow

(A) Immunostaining showing as indicated the labelling of isolated sca-1$^+$ cells (Scale bars: 50 μm).

(B) Quantitative analysis of qPCR showing mRNA expression of SMC markers (Acta2, Tagln and Myh11) in TGFβ1 treated Sca-1$^+$ VPCs. All the data represent mean ± SEM, n=3 experiments.

(C) Representative immunostaining images showing expression of SMC markers (α-SMA and SM22) in TGFβ1 treated Sca-1$^+$ VPCs. Images shown are representative of n=3 separate experiments.

(D, E) Heatmap showing expression of specific genes of transcription factors and ECM production along the sc-RNAseq pseudotime trajectory of mixing cells including smooth muscle cells and adventitial cells in vein grafts.

(F) Volcano plot showing bulk RNA-seq of Sca-1$^+$ VPCs and Sca-1$^+$ BMCs. Each dot labeled in the plot represented a gene that is related to the vascular development.

(G) Biological function analysis showing different functions of enriched genes in Sca-1$^+$ VPCs and Sca-1$^+$ BMCs.

Abbreviations: Acta2, Actin α2, Smooth Muscle; α-SMA, α-Smooth Muscle Actin; BMC, bone marrow cell; Bulk RNA-seq, Bulk RNA sequencing; ECM, extracellular matrix; My11, Myosin heavy chain 11; qPCR, quantitative polymerase chain reaction; SM22, Transgelin; sc-RNAseq, single cell RNA sequencing; Tagln, Transgelin; VPC; vascular progenitor cell.
